# HIV-1 compartmentalization in the central nervous system during cryptococcal meningitis: Analysis of viral, fungal, and host factors

**DOI:** 10.1371/journal.ppat.1014697

**Published:** 2026-10-15

**Authors:** Maura Manion, Camille M. Lange, Ana M. Ortega-Villa, Christine M. Fennessey, Brandon F. Keele, Matthew Moeser, Helene Highbarger, Kristi Huik, Natalie Lindo, Christian Gonsalves, James Virga, David B. Meya, Sarah B. Joseph, David R. Boulware, Irini Sereti, Frank Maldarelli

**Affiliations:** 1 Intramural Clinical Management and Operations Branch, Office of the Clinical Director, National Institute of Allergy and Infectious Disease (NIAID), NIH, Bethesda, Maryland, United States of America; 2 Clinical Retrovirology Section, HIV Dynamics and Replication Program, National Cancer Institute, National Institutes of Health, Frederick, Maryland, United States of America; 3 HENYIDA LLC, Lewes, Delaware, United States of America; 4 Office of Biostatistics Research, National Institute of Allergy and Infectious Diseases, National Institutes of Health, Bethesda, Maryland, United States of America; 5 AIDS and Cancer Virus Program, Frederick National Laboratory for Cancer Research, Frederick, Maryland, United States of America; 6 Department of Microbiology and Immunology, University of North Carolina, Chapel Hill, North Carolina, United States of America; 7 Virus Isolation and Serology Laboratory, Frederick National Laboratory for Cancer Research, Frederick, Maryland, United States of America; 8 Department of Medicine, School of Medicine, College of Health Sciences Makerere University, Kampala, Uganda; 9 Department of Medicine and International Health, University of Minnesota, Minneapolis, Minnesota, United States of America; 10 Laboratory of Immunoregulation, Division of Intramural Research, National Institute of Allergy and Infectious Disease (NIAID), NIH, Bethesda, Maryland, United States of America; University of Illinois at Chicago College of Medicine, UNITED STATES OF AMERICA

## Abstract

Opportunistic infections in the central nervous system (CNS), such as cryptococcal meningitis (CM) have profound consequences for people living with HIV-1 (PWH), including blood-brain barrier impairment and neuroinflammation. To investigate how CM may shape the HIV-1 populations in the CNS, we characterized viral variants in the blood and cerebrospinal fluid (CSF) compartments in PWH with active CM. Thirty-nine participants with CM had paired plasma and CSF viral load measurements prior to the start of anti-retroviral therapy (pre-ART), with overall significantly higher HIV-1 viral loads in plasma compared to the CSF. Among those with CSF pleocytosis, CSF viral loads were relatively higher and CCR5 coreceptor-utilizing HIV-1 were more frequently detected. To investigate whether these differences were due to sustained HIV-1 replication in the CNS that generated genetically distinct viral lineages in the CSF (i.e., viral compartmentalization), we characterized pre-ART HIV-1 *env* populations in a subgroup of eighteen PWH with CSF HIV-1 RNA levels > blood (N = 9) and CSF RNA levels < blood (N = 9). Compartmentalization between CSF and plasma viral populations was detected in participants from both groups and was associated with higher interleukin-2 (IL-2) levels in CSF. In some cases, HIV-1 populations in CSF were compartmentalized from plasma, were exclusively macrophage-tropic and reached levels exceeding 10^5^ copies/ml CSF. In other cases, HIV-1 infected cells trafficking across the plasma and CSF compartments seemed the most likely source of virus in the CSF. Post-ART analysis in one participant demonstrated emergence of distinct HIV-1 populations. This study demonstrates that opportunistic infections may have diverse effects in shaping HIV-1 viral populations.

## Author summary

Cryptococcal meningitis (CM) is an infection that disrupts the blood-brain-barrier, providing a useful model to investigate host and fungal factors influencing HIV-1 populations in this critical anatomic compartment. In this study, we assessed viral characteristics (e.g., HIV-1 subtype, levels of viral RNA in CSF and blood, *env* entry phenotype), fungal attributes (cryptococcal antigen levels) and host features (soluble and cellular immune biomarkers) to determine their relationship to HIV-1 compartmentalization during cryptococcal meningitis. We studied paired plasma and cerebrospinal fluid (CSF) in a Ugandan cohort prior to initiation of ART, including a substantial proportion of PWH with highly pathogenic HIV-1 Subtype D. We found that HIV-1 compartmentalization during CM and prior to ART initiation (pre-ART) was associated with elevated CSF interleukin-2. Compartmentalized HIV-1 in the CSF may consist of macrophage-tropic HIV-1. We also studied one participant who initiated and then stopped ART; although the genetic composition of their rebound plasma viremia was related to pre-ART CSF and blood populations, the post-ART CSF rebound viruses were genetically distinct. These findings suggest that cryptococcal meningitis may influence CNS HIV-1 populations prior to ART initiation, potentially impacting the dynamics of viral reservoirs.

## Introduction

Human immunodeficiency virus (HIV)-1 results in widespread infection of lymphoid and non-lymphoid tissues, including the central nervous system (CNS) [1–4]. The CNS is also a site for several opportunistic infections, such as cryptococcal meningitis, John Cunningham virus and tuberculosis, which result in considerable morbidity and mortality. Antiretroviral therapy (ART) successfully suppresses HIV-1 viremia, increases CD4 cell numbers, and dramatically improves survival which can now approximate that of HIV-1-seronegative people [5]. ART blocks ongoing viral replication, but it does not eradicate HIV-1 infected cells, allowing persistent anatomic reservoirs, such as the CNS, to contribute to ongoing neurocognitive dysfunction during treatment [6,7]. The mechanisms that underlie HIV-1 infection in the CNS remain poorly understood; clarifying these processes is essential to understanding how these reservoirs persist despite ART in order to develop targeted cure strategies. Host susceptibility to HIV-1 infection, intrinsic virologic characteristics, opportunistic infections that compromise the blood brain barrier (BBB) and localized immune responses may collectively influence pre-ART HIV-1 replication rates, viral load levels and the establishment of viral reservoirs upon ART initiation.

HIV-1 DNA and RNA have been detected in cerebrospinal fluid (CSF) and brain tissue of people living with HIV-1 (PWH) on ART [1–3,8–12]. We recently demonstrated that the CNS could serve as a site of HIV-1 production, leading to viral rebound [10]. HIV-1-infected cells can traffic to the CNS during acute infection [13,14] and persist in long-lived CNS-resident macrophage-like cells (e.g., microglial cells), which are susceptible to HIV-1 infection [12]. HIV-1 RNA levels in cerebrospinal fluid (CSF) are typically a log, or more, lower than levels in peripheral blood, but may occasionally exceed plasma levels [10,15]. HIV-1 populations in CSF or brain-derived tissue are genetically diverse and may include macrophage-tropic variants [12]. Therefore, viral populations in the CNS may be distinct from, or resemble those in peripheral blood, but the factors driving their compartmentalization remain unclear. In chronic HIV-1 infection, HIV-1 populations are frequently equilibrated between the blood and CSF prior to ART initiation, indicative of HIV-1-trafficking across the BBB [11]. Furthermore, compartmentalization of CSF and peripheral HIV-1 populations can be driven by expansion of T cell-tropic and macrophage-tropic virus during acute infection [16,17].

Cryptococcal meningitis (CM) due to *Cryptococcus neoformans* (*C. neoformans*) is the most common fungal CNS infection in PWH, accounting for ~19% of AIDS-related deaths [18] with a median mortality of 31.6% within two years following diagnosis [19]. Approximately 4% of PWH with CD4 counts ≤200 cells/µL have cryptococcal antigenemia, and 70–90% of those who are untreated or not linked to care will progress to symptomatic meningitis [18]. This equates to an estimated 152,000 cases of cryptococcosis in PWH each year, denoting a significant impact of cryptococcal disease on PWH.

CM disrupts BBB integrity and promotes recruitment of effector T cells into the CNS [20,21]. Trafficking of activated immune cells across a compromised BBB has been implicated in shaping HIV-1 populations as infection persists [10,22,23]. Distinct CSF HIV-1 populations have been observed with comorbidities such as CM, suggesting that BBB disruption does not necessarily prevent compartmentalization [24]. However, studies of CNS compartmentalization remain limited, particularly as it pertains the role of macrophage-tropic HIV-1 and rapidly progressing subtypes (e.g., subtype D) [24]. The immune mechanisms that drive seeding of CNS HIV-1 reservoirs remain poorly understood; analyses of immune responses, including cytokine profiles, have not shown consistent associations with compartmentalization, although viral populations have been linked to distinct immune signatures [10,25,26]. Additionally, the contribution of macrophage-tropic HIV-1 during active CM remains unknown.

We hypothesized equilibrated viral and proviral HIV-1 populations in blood and CSF as a result of a severely compromised BBB during chronic HIV-1 infection and active CM. To investigate the role of CM on non-subtype B HIV-1 populations in the CNS, we analyzed virological and fungal characteristics, as well as immune responses in PWH with *C. neoformans* associated CM in South Africa and Uganda (NCT01075152 and NCT01802385). Thirty-nine participants had paired plasma and CSF samples available for HIV-1 viral load measurements. We used phylogenetic analyses of single genome HIV-1 envelope sequences (*env)* to characterize pre-ART viral and proviral populations in peripheral blood and CSF of 18 participants with HIV-1/*C. neoformans* coinfections.

## Results

### Participant characteristics

Plasma and CSF samples were available for 39 study participants with HIV-1 and CM prior to ART from the Cryptococcal Optimal ART Timing (COAT) and Adjunctive Sertraline for Treatment of Cryptococcal Meningitis (ASTRO-CM) trials. The median participant age was 37 years old, 33% were female and the median CD4 count was 17 cells/µL (Table 1). In terms of outcomes, eight of the 39 participants died and sixteen developed immune reconstitution inflammatory syndrome (IRIS). The median plasma viral load (VL) was 5.2 log_10_ copies/mL [IQR 4.5, 5.6], while CSF VL was 4.8 log_10_ copies/mL [IQR of 4.0, 5.3], with a median CSF-to-plasma ratio of 0.92 [IQR 0.83, 1.1] (Fig 1A, 1B). This range of viral RNA levels was similar to previous reports of PWH with CM [15,27–29]. Overall CSF VL was statistically significantly lower than plasma VLs (Table 1), but the difference was < 0.5 log, in sharp contrast to the ≥ 1 log difference typically observed in PWH without pre-ART CNS co-infections [6,28,30]. Notably, 14/39 participants (36%) had CSF VL higher than plasma VL (Table 1). A higher CSF VL compared to plasma VL was not associated with differences in demographic (Table 1), however it was associated with a lower plasma viremia and was more common with CSF pleocytosis (CSF White cell count [WCC] ≥5) (Fig 1C), consistent with previous reports [6].

**Table 1:** Baseline characteristics of 39 participants with HIV-1 and cryptococcal meningitis.

Variables	CSF Viral Load ≥ Plasma Viral Load	CSF Viral Load <Plasma Viral Load	Overall	P-value
N	N = 14	N = 25	N = 39	
Age, years	35 [28, 47]	37 [29, 42]	37 [29, 43]	0.988
Female Sex	8 (43%)	7 (28%)	15 (33%)	0.555
CD4 cells/µL	40 [17, 75]	12 [7, 69]	17 [9, 70]	0.164
CSF White cells/µL	<5 [<5, 50] (N = 13)	<5 [<5, 25]	<5 [<5, 33] (N = 38)	0.790
CSF White cells <5/µL^a^	8 (62%)	16 (64%)	24 (63%)	0.999
CSF Total Protein, mg/dL	88 [48, 147] (N = 12)	83 [59, 141]	85 [53, 141] (N = 37)	0.846
CSF Opening Pressure, mm H_2_O	360 [253, 533]	340 [222, 440]	360 [230, 465]	0.703
CSF *Cryptococcus* Culture, log_10_ CFU/mL CSF	4.9 [4.1, 5.6] (N = 13)	5.1 [4.6, 5.5]	5.1 [4.4, 5.6] (N = 38)	0.612
CSF HIV-1 log_10_ copies/mL	5.4 [5.0, 5.5]	4.4 [3.8, 5.1]	4.8 [4.0,5.3]	0.001
Plasma HIV-1 log_10_ copies/mL	4.9 [4.4, 5.2]	5.5 [4.9, 5.6]	5.2 [4.5,5.6]	0.013
Paradoxical IRIS	5 (36%)	11 (44%)	16 (41%)	0.869
Death	2 (14%)	6 (24%)	8 (21%)	0.759

Data are N (%) or median (IQR). P-values from chi-squared or Mann Whitney tests as appropriate. Specified N denotes instances where the full dataset was unavailable. ^a^ The number and percentage of CSF White Cells from the row above that are < 5/µL.

**Fig 1:**
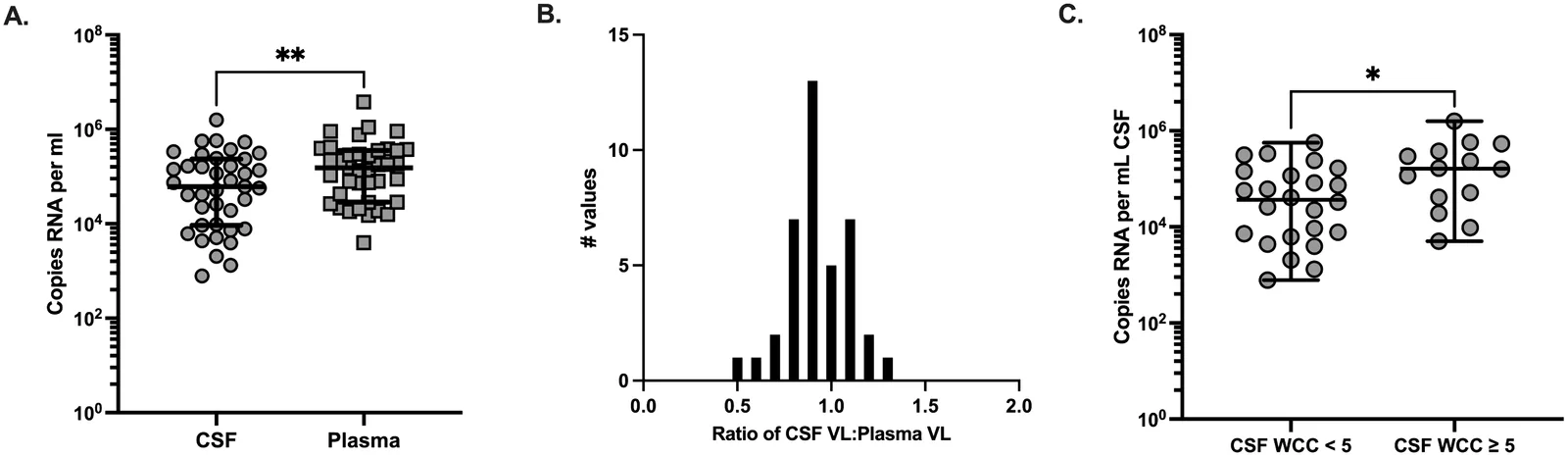
CSF and plasma HIV-1 viral loads during cryptococcal meningitis. **A.** HIV-1 viral loads were overall higher in plasma compared to CSF. Median and IQR are shown. Comparison by paired Wilcoxon signed rank test (p = 0.007)**. B.** Histogram of ratio of CSF:plasma viral loads **C.** Study participants with CSF pleocytosis during CM had higher CSF viral loads compared to those with CSF WCC < 5 cells/mL. Median and IQR are shown. Comparison by unpaired Mann-Whitney test (p = 0.027).

Study participants presented with high burdens of cryptococcal disease with a median quantitative cryptococcus culture of 5.1 log_10_ CFU/mL and a median intracranial opening pressure of 360 mm H_2_0 (Table 1). The median neurocognitive performance z-score (QNPZ) at the end of study was -1.055, similar to prior studies [31]. No significant differences in neurocognitive scores or biomarkers were observed among PWH with higher versus lower CSF VL relative to plasma VL (S1A Fig and S1 Table, respectively), indicating that elevated CSF VL levels were not associated with impaired CNS function nor altered soluble immune responses.

HIV-1 *env* sequences from CSF and plasma were genetically characterized in a subgroup of 18 participants: 9 with higher CSF VL levels than plasma and 9 with higher viral loads in the plasma. Characteristics for these 18 study participants are shown in S2 Table. Notably, 44% of participants were female, 33% died (N = 6), and 67% (N = 12) developed IRIS during their clinical course. The majority of this subgroup were infected with HIV-1 subtypes D (9 participants) or A1 (7 participants), with one participant each infected with a recombinant AD subtype and subtype C. Plasma and CSF HIV-1 subtypes matched in all participants except participant 18, whose plasma subtype was A1 while the CSF contained both A1 and C (S2 Table). No differences in neurocognitive scores were found between participants infected with subtype D compared to those with non-subtype D infections (S1B Fig).

Single genome sequencing was used to investigate genetic diversity, coreceptor utilization and population dynamics in both the CSF and plasma compartments. Overall diversity of HIV-1 populations in the CSF and plasma during CM was substantial and not significantly different (APD range 0.01-0.05, S3Fig), suggesting extensive viral replication in both compartments. Phylogenetic analysis revealed that viral populations in each participant were distinct from those in the other participants (S2 Fig and S1 Appendix), confirming the absence of cross-contamination in the sequencing process. Several study participants had diverse trees with large branches with strong bootstrap support (e.g., P2, P4, P18). The etiology of these topologies is unclear; they may reflect multiple rounds of infection, although the phylogenies lack intermediary branches indicative of recombination and with overall diversity comparable to other participants.

### HIV-1 population diversity and composition

To compare viral populations in plasma and CSF compartments, we analyzed the genetic diversity and composition of HIV-1 *env* in each compartment. The average number of single genome sequences obtained per participant was 22 from CSF and 21 from plasma (S3 Table), providing 95% probability of detecting *env* variants at a frequency of ≥10% in plasma, and representative of an adequate sampling to measure viral diversity and detect minority species within the population [4,32]. Plasma and CSF HIV-1 populations had similar genetic diversity (mean difference = 0.004 or 10 nucleotides, S3 Fig). However, several PWH had relatively few distinct variants in CSF, and overall, the distribution of populations was distinct, with a modestly higher proportion of unique variants in plasma than in CSF (Fig 2, p = 0.030, Wilcoxon paired rank). These data suggest that in some cases, high HIV-1 RNA levels in CSF were driven by only a few variants, whereas many distinct variants contributed to high plasma HIV-1 RNA levels.

**Fig 2:**
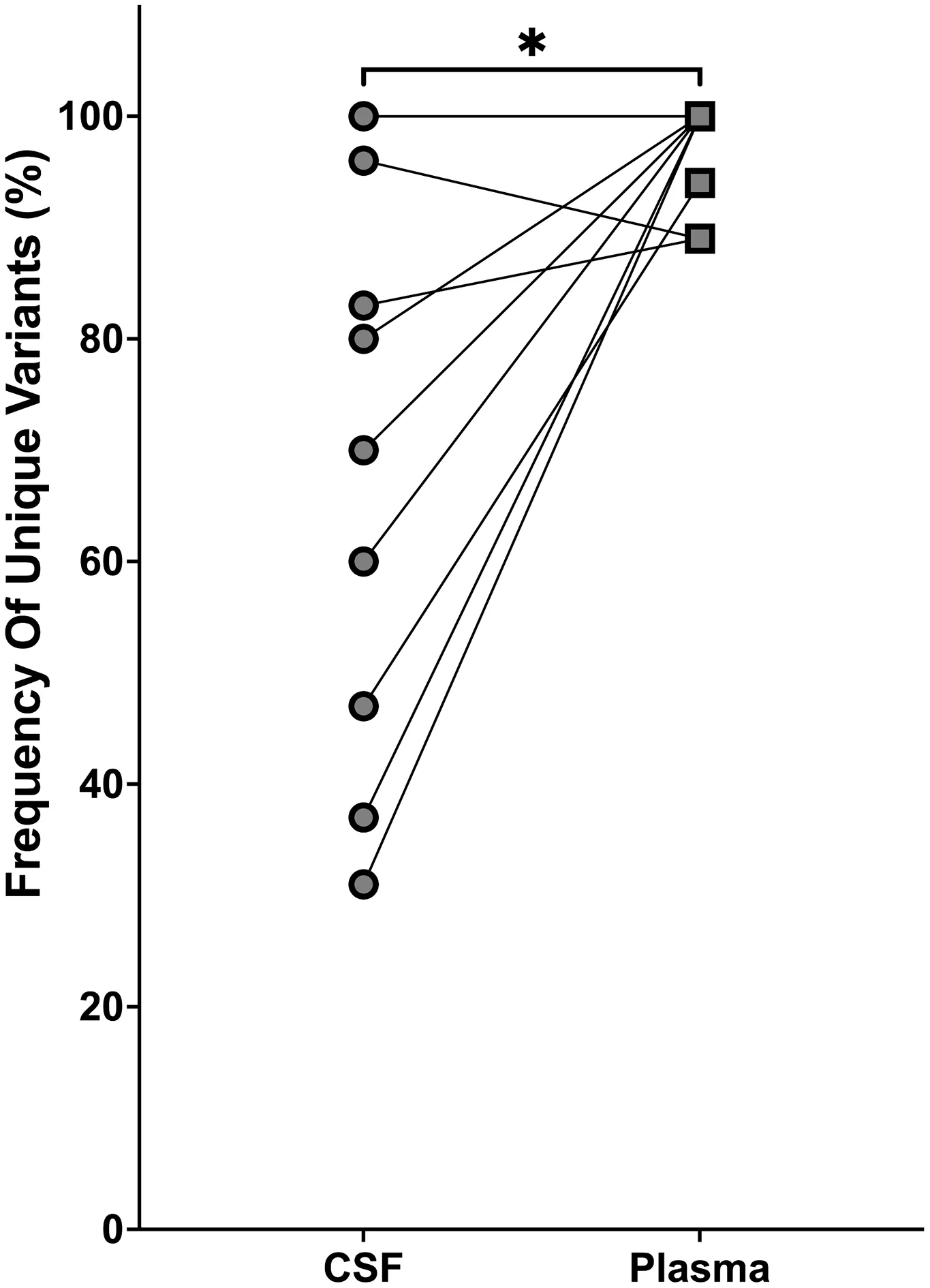
Diversity of CSF versus plasma HIV-1 populations during cryptococcal meningitis. Higher abundance of unique variants existed in plasma than CSF. Data points in the plasma are superimposed. Comparisons done by Wilcoxon signed rank test (p = 0.030).

### HIV-1 co-receptor utilization in the CSF and plasma

We performed in silico analyses of HIV-1 V3 sequences to predict co-receptor usage (CCR5 vs CXCR4) in CSF and plasma. Sequences with a false positive rate (FPR) <2.6% were considered CXCR-4 utilizing virus [33,34]. Participants with CM were predicted to harbor both CCR5- and CXCR4-utilizing viruses in both compartments (S4 Table); approximately 40% had exclusively CCR5-utilizing viruses in both their CSF and plasma. Infrequently, CXCR4- utilizing viruses predominated in plasma while CCR5-utilizing viruses were more common in CSF (S4 Table, P1, P2, P4, P5). These data suggest limited partitioning of blood and CSF, likely reflecting BBB disruption during CM-related inflammation and increased trafficking of HIV-1 into CSF compartments [11,21]. Furthermore, those with elevated CSF WBC counts (WBC > 5 cells/µl) had CCR5-tropic viruses in CSF (FPR ≥ 2.6%, Fig 3; Mann-Whitney p = 0.018). Within this group, one participant (P5) had both X4- and R5-tropic HIV-1; 50% of their plasma virus was X4-tropic (FPR < 2.6). These findings suggest a complex relationship between HIV-1 population in blood and CSF.

**Fig 3:**
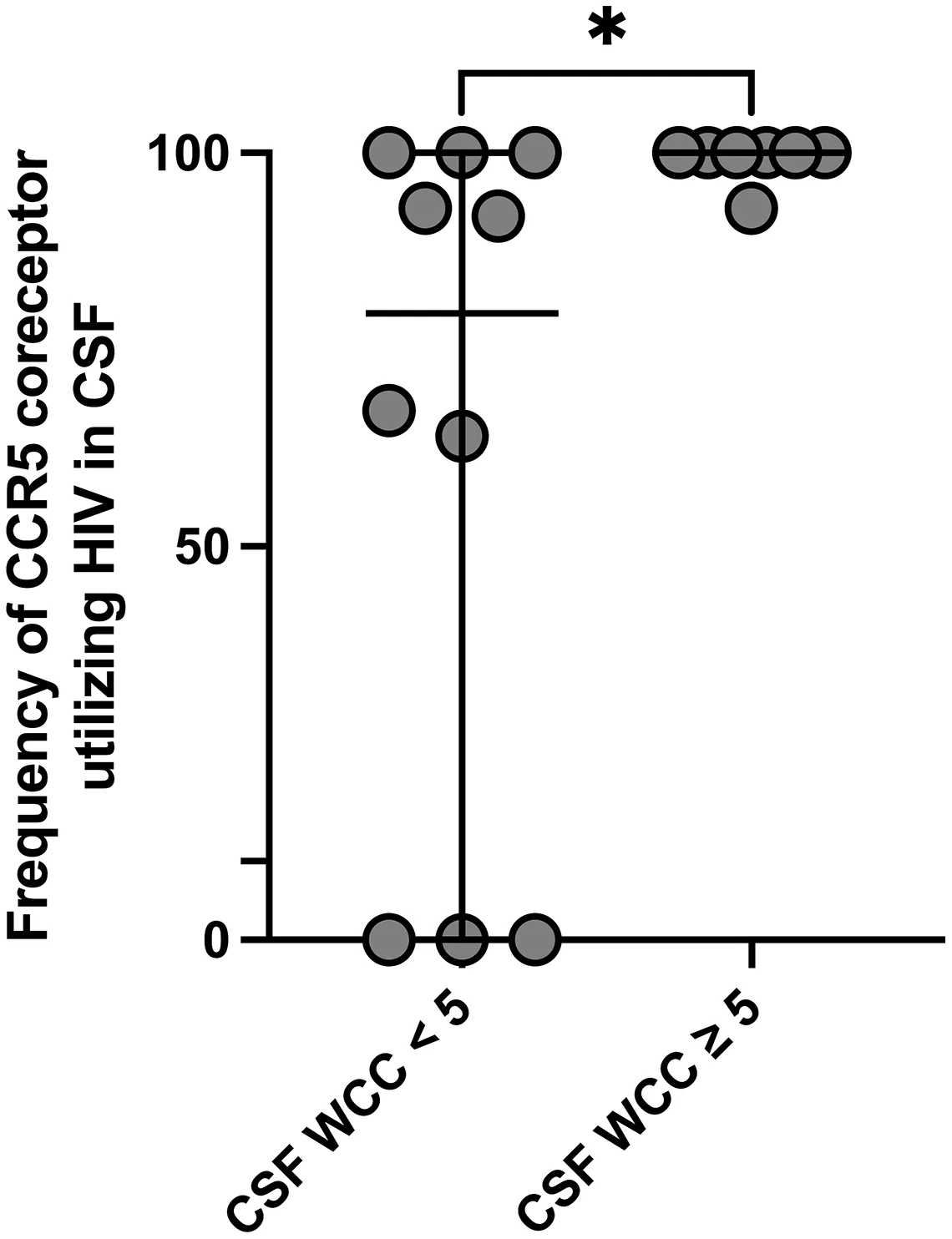
Frequency of CCR5/nonCXCR4 coreceptor utilizing HIV-1 in the CSF. Higher frequencies of CCR5 coreceptor utilizing HIV-1 in the CSF were observed in PWH with CSF pleocytosis. CCR5 co-receptor utilization of *env* sequences was determined using a series of false positive cutoff of <2.6. Median and IQR are shown. Comparisons by Mann-Whitney test (p = 0.018).

### Pre-ART compartmentalization of HIV-1 RNA populations between CSF and blood

To investigate the relative distribution of HIV-1 variants in CSF and blood and identify other potential mechanisms responsible for composition of HIV-1 populations in CSF, we conducted a series of population genetic and phylogenetic compartmentalization studies. First, we used direct non-parametric tests of compartmentalization [35] to analyze blood and CSF HIV-1 RNA populations for all 18 participants. Seven participants (39%) had strong evidence that HIV-1 populations in CSF were compartmentalized from the plasma (Fig 4A). To determine if this viral compartmentalization was simply due to over-representation of identical sequences, we repeated the analyses with unique variants only (i.e., identical sequences were represented by a single sequence). We also used a parametric phylogenetic-based compartmentalization analysis (Slatkin-Maddison) to validate non-parametric testing outcomes [36].

**Fig 4:**
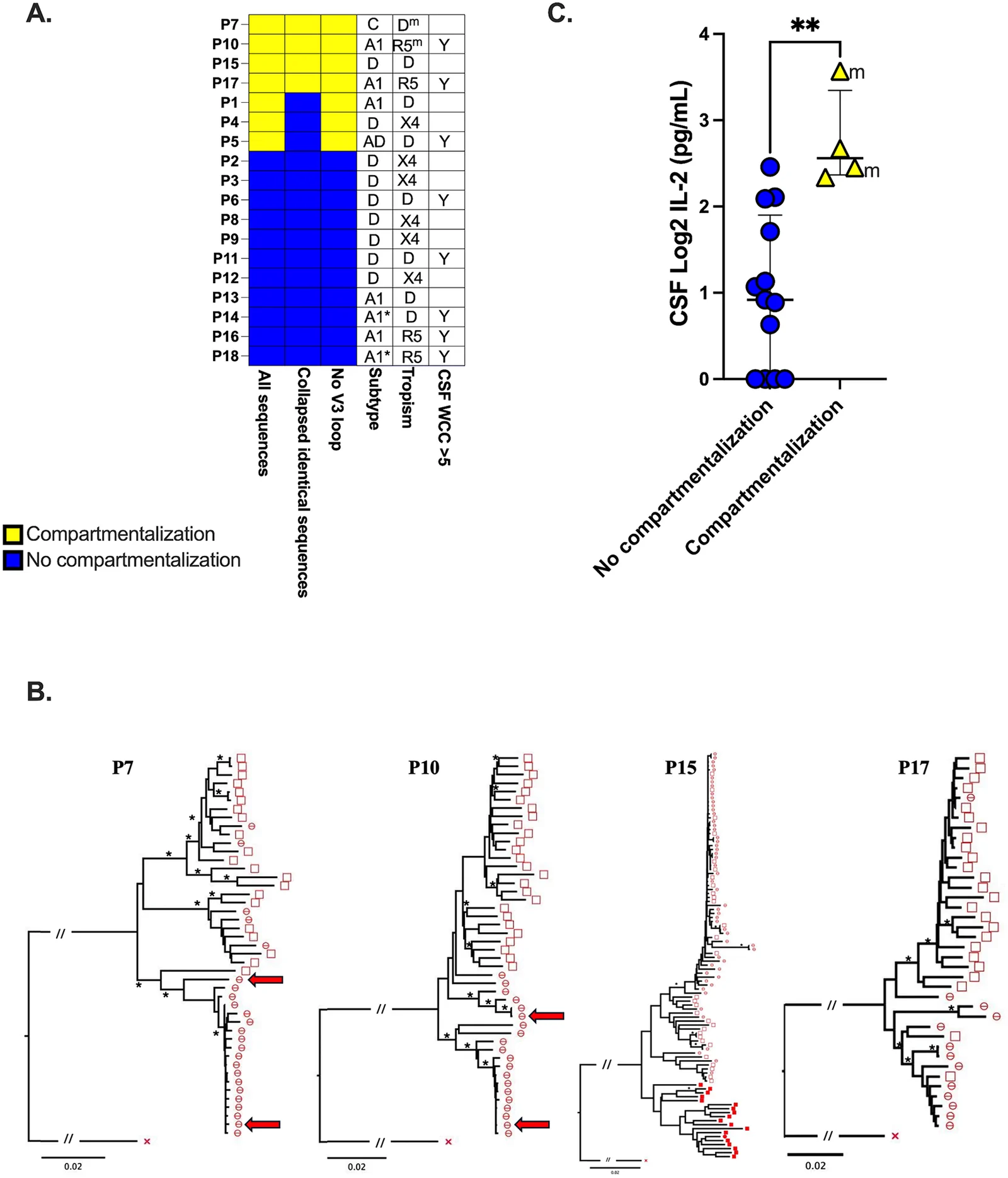
Compartmentalization of CSF and plasma HIV-1 populations. **A.** The role of identical sequences and coreceptor utilization in PWH with compartmentalization. Sequence sets were analyzed using non-parametric and parametric analyses [35,36]. Participants with evidence of compartmentalization in both parametric and non-parametric analyses are depicted with yellow squares; comparisons without evidence of compartmentalization in both tests are depicted in blue. *In the subtype column, P18 is 71% A1 and the rest of populations are subtype C. In the tropism column, D indicates dual-tropic virus; P7 and P10 were found to have macrophage tropic virus, denoted D^m^ and R5^m^, respectively. **B.** Phylogenetic trees of the HIV-1 RNA populations of the 4 participants with compartmentalization of CSF (circles) and plasma compartments (square); filled symbols indicate X4-tropic viruses. Stars are branches with boot strap values >99. Red arrows indicate Macrophage-tropic variants identified in CSF**. C)** CSF IL-2 levels are higher in participants with compartmentalization between CSF and plasma. Median and IQR are shown. Comparisons by Mann-Whitney test (p = 0.002).

Over-representation of identical viral sequences drove compartmentalization of CSF and plasma viruses in 3 of 7 participants (Fig 4A). Removal of the V3 loop from viral populations did not change compartmentalization patterns, indicating that CCR5-tropism alone does not explain viral compartmentalization across the BBB. Phylogenetic analyses showed strong bootstrap support separating CSF- and blood-derived HIV-1 sequences, even when >95% of variants were R5-tropic (Fig 4B, S4 Table). Taken together these data indicate that HIV-1 populations in the CSF of P7, P10, P15 and P17 were compartmentalized despite active CM. The mechanisms underlying this compartmentalization remain unclear. It is unknown why CSF-specific variants are absent from plasma, or why plasma variants appear unable to access the CSF, and whether this reflects establishment of a CNS reservoir prior to CM infection. Several participants (P1, P4 and P5) had five or fewer unique CSF HIV-1 sequences, limiting the robustness of compartmentalization analyses. Therefore, the true frequency of compartmentalization may be higher than observed in this study.

We did not find an association of compartmentalization in participants with the more pathogenic subtype D virus, tropism, or CSF pleocytosis (Fig 4A). In addition, we did not find an association of compartmentalization with the outcomes of IRIS or death. Two participants (P10, P15) developed IRIS, one expired (P10), while P7 and P17 did not experience IRIS and both survived (S2 Table). We did find, however, significantly higher CSF IL-2 levels in individuals with compartmentalization of viral sequences in the CSF and plasma (Fig 4C and S5 Fig) regardless of the presence of elevated WCC. It is unclear whether elevated levels of IL2 have a role in driving compartmentalization or represent a consequence of R5 viruses present in the CNS.

To further characterize these compartmentalized CCR5-utilizing variants in CSF, we used the well-established affinofile assay to measure their ability to efficiently enter cells expressing a low density of CD4 (similar to that expressed on macrophages) as a proxy for macrophage tropism [37]. We analyzed CSF variants from compartmentalized viral populations (P7, P10, P15, P17) and clearly non-compartmentalized populations (P2). As shown in Fig 5, *env* sequences from compartmentalized CSF lineages in P7 and P10 exhibited strong macrophage tropism, whereas variants from P15 and P17 showed intermediate or no macrophage tropism. CSF variants from P2 and P18 exhibited no macrophage tropism. CSF HIV-1 RNA levels in P7 and P10 were relatively high and the variants assessed for macrophage tropism represented most or all sequences obtained from the CSF compartment CSF (Fig 4B). Therefore, in these participants, the majority of the CSF viral population (exceeding 10^5^ copies/mL) consisted of macrophage-tropic viruses. Macrophage-tropic populations were observed with and without high WCC counts (S2 Table), and in the presence of relatively high IL-2 levels in compartmentalized populations (Fig 4C). Macrophage-tropic viruses were absent from the CSF of other participants with compartmentalized virus (Fig 5), demonstrating that T cell-tropic HIV-1 can replicate in the CNS and form compartmentalized lineages.

**Fig 5:**
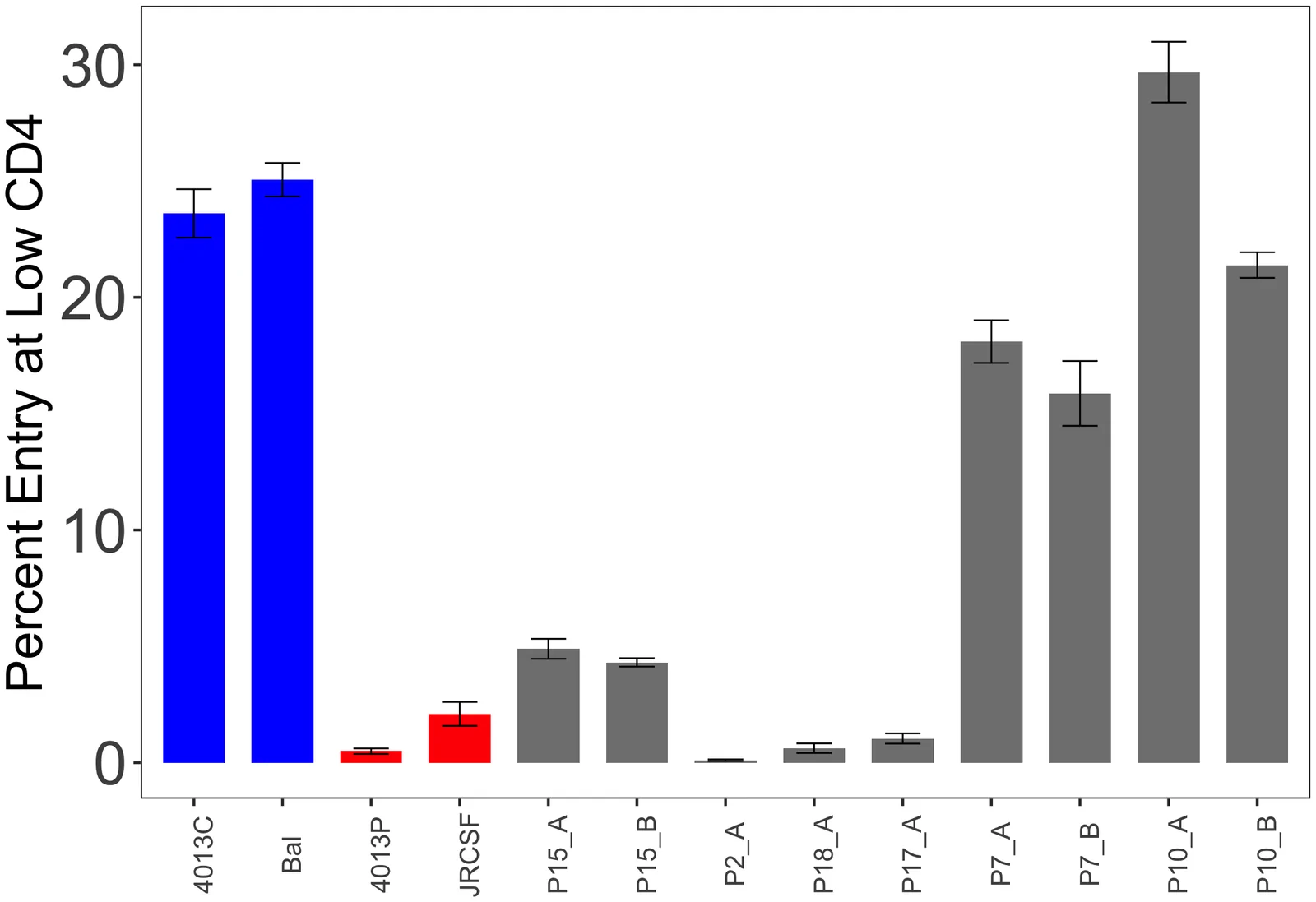
Some compartmentalized CSF variants are Macrophage-tropic. CSF derived HIV-1 *env* sequences from the indicated participants were cloned into expression plasmids and transfected into 293 cells. Resulting pseudoviruses were harvested from medium and ability to infect low CD4 expressing cells was evaluated in affinofile cells as previously described [37], and expressed as percent entry at low CD4.

### Pre-ART compartmentalization of cell-associated HIV-1 RNA and DNA populations between CSF and blood

To investigate cell-associated HIV-1 and identify sources of HIV-1 in CSF and plasma, we performed single genome analyses from HIV-1 populations derived from paired plasma, PBMCs, CSF and CSF cells that were available for 5 of 18 participants (S3 Table). The average number of sequences per participant was 22 from CSF RNA, 21 from plasma RNA, 17 from CSF DNA and 24 from PBMC DNA, providing a 95% probability of detecting *env* variants at a frequency ≥15% [4]. We performed parametric and non-parametric analyses to determine compartmentalization of HIV-1 infected cell populations during CM. HIV-1 DNA populations in CSF and PBMC were not compartmentalized in any of the five participants (S4 Fig), suggesting substantial trafficking of infected cells across compartments, consistent with prior reports in HIV-1/HCV coinfected individuals [11]. CSF RNA populations were a mixture of lineages observed in CSF DNA and PBMC DNA, indicating that infected cells from both compartments contribute to virus in the CSF compartment in these participants during CM. In contrast, P15 showed compartmentalization between CSF and plasma HIV-1 RNA populations, despite a lack of compartmentalization between HIV-1 DNA and RNA populations in CSF and plasma (S4 Fig and Fig 4A). In this participant, most CSF virus utilized CCR5 for viral entry, whereas the plasma consisted of both CCR5- and CXCR4-utilizing variants. In P2, PBMC HIV-1 DNA populations were compartmentalized from both plasma and CSF HIV-1 RNA populations, although CSF and plasma RNA populations were mixed. Phylogenetic analysis of P2 (S1 Appendix) demonstrated that only a minority of infected PBMCs contributed to CSF and plasma virus populations.

The discordance between viral DNA and RNA HIV-1 populations likely reflects the different sources of the derived sequences, i.e., HIV-1 DNA amplified from PBMC includes large numbers of defective proviruses that accumulate during prolonged infection in PWH with advanced HIV-1/AIDS. In contrast, RNA sequences likely represent virus from a smaller population of transcriptionally active proviruses from recent viral replication cycles. Consistent with these assumptions, most *env* sequences recovered from RNA were genetically intact. Differences in compartment dynamics are also contributing factors; CSF replenishment is relatively slow compared to continuous plasma replenishment, therefore virion clearance in CSF may occur at rates distinct from the trafficking of infected cells from peripheral circulation. Longitudinal studies of HIV-1 dynamics and lymphocyte trafficking across anatomic compartments will be needed to clarify these contributing mechanisms.

### Evaluation of biomarker profiles in the CSF

We evaluated soluble biomarker profiles in 6 participants with paired CSF and plasma cytokine/chemokines measurements to determine immune factors associated with HIV-1 compartmentalization between blood and CSF during CM. As noted previously, elevated levels of IL-2 in CSF were associated with compartmentalization between CSF and blood viruses (Fig 4C and S5 Fig). We found distinct immune signaling patterns in each compartment during CM. CSF showed higher level of granulocyte-macrophage colony-stimulating factor (GM-CSF), Monocyte chemoattractant protein -1 (MCP-1), IL-2, IL-8, and IL-10, while plasma had higher levels of MIP-1B and IL-7. These compartment-specific biomarker profiles during CM suggest differential immune activation and chemokine signaling, particularly involving altered levels of MCP-1, IL-8, MIP-1B which regulate immune cell trafficking (S6 Fig) [38,39]. Furthermore, the presence of identical viral sequences was more common in participants with lower IFN-γ, TNF-α, and IL-4 levels (S7 Fig). CD4 count, viral loads, opening pressure at lumbar puncture, CSF profile and other CSF biomarkers did not differ based on evidence of compartmentalization or the presence of identical sequences.

### Post-ART compartmentalization of HIV-1 RNA populations

HIV-1 RNA levels in the CSF and plasma typically undergo rapid suppression upon initiating ART, but non-adherence and discontinuation can lead to viral rebound. Furthermore, the origin and fate of rebound CSF HIV-1 variants are not well understood. One study participant, P6, initiated and subsequently discontinued ART, which led to significant viral rebound in plasma. We analyzed viral populations in plasma and CSF collected 85 and 98 days after ART discontinuation, respectively (Fig 6), at the time of cryptococcal relapse and HIV-1 viral rebound. P6’s pre-ART viral populations across both compartments were mixed and predominantly CXCR4-utilizing. However, CSF virus detected 98 days post-ART, was genetically diverse and phylogenetically distinct from all pre-ART CSF and plasma populations, (Fig 6) as well as post-ART plasma populations. Notably, the complete glycoprotein 41 (gp41) sequence could not be recovered from this post-ART sample, limiting macrophage-tropism analyses. Multiple sequencing attempts failed to amplify the final 168 nucleotides of gp41, likely due to mis-priming. We cannot rule out, the possibility of a variant with an authentic truncation in gp41, present in all viral variants detected at 98 days post-ART, was selected when ART was initiated. A presumed deletion would be very similar to a deletion in gp41 previously observed after in vitro cultivation, in which virus with the deletion was shown to replicate effectively in macrophages *in vitro* [40]. Notably, the emergence of truncated CSF variants could account for the profound shift in the CSF HIV-1 population after ART initiation; in contrast, all of P6’s plasma-derived HIV-1 *env* variants throughout and post-ART contained full length gp41. Taken together, these data suggest a potential scenario where rebound HIV-1 in CSF may arise from a large reservoir of proviral populations that established infection in the CNS.

**Fig 6:**
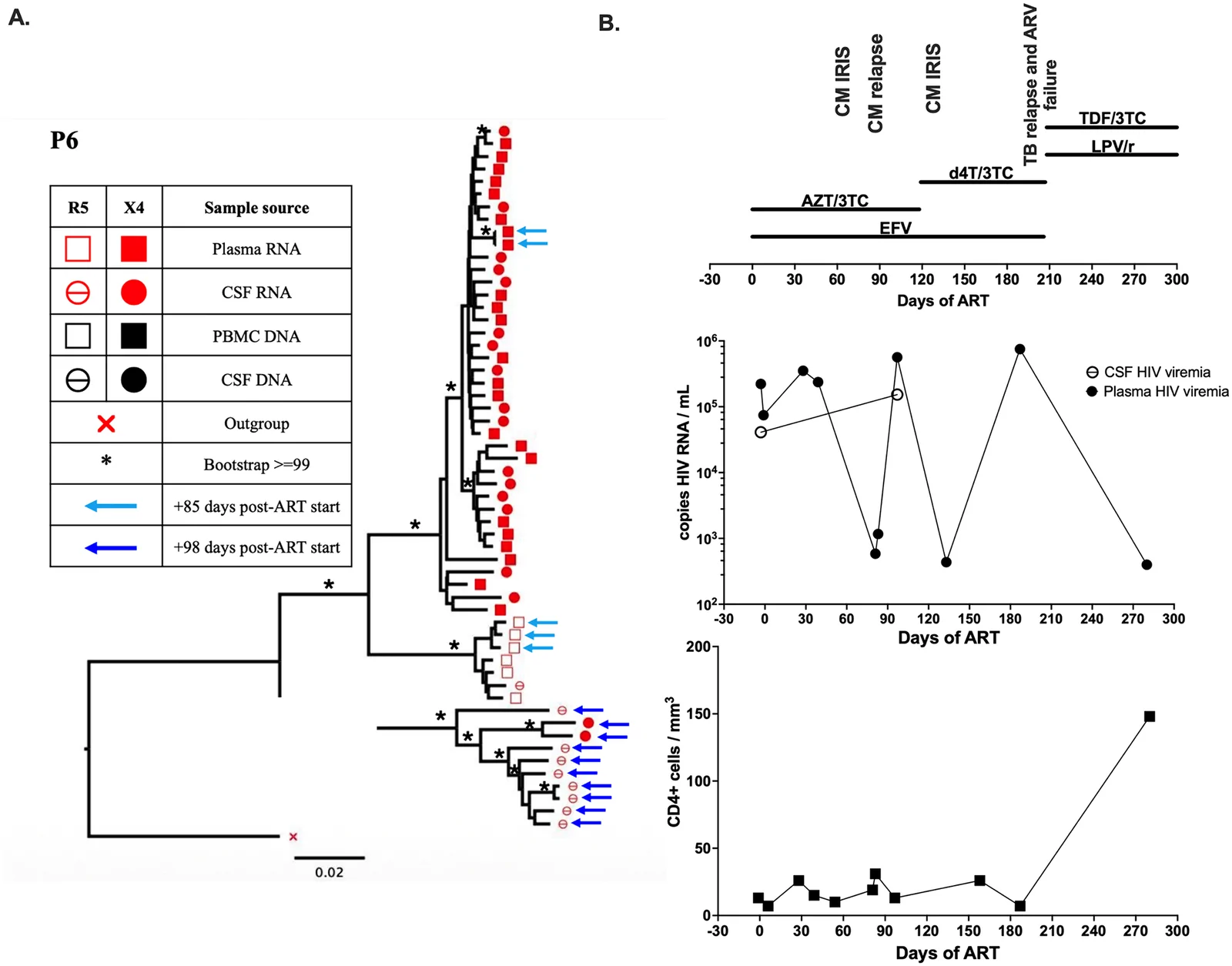
HIV-1 population shift after rebound viremia. **A.** Phylogenetic tree of P6. Pre-ART CSF and plasma are panmictic. CSF HIV-1 populations after 98 days of ART are compartmentalized from other HIV-1 populations. HIV-1 symbols as per the legend on the figure. **B.** P6 HIV-1 RNA viral load and CD4 count timelines with therapies and interventions indicated.

## Discussion

Through evaluation of CSF and plasma viral populations in a cohort of participants with HIV-1 and CM prior to ART, our goal was to gain a better understanding of the mechanisms driving establishment and persistence of the CNS HIV-1 reservoir during CNS opportunistic infections [41–43]. We hypothesized that CM associated disruption of the BBB would permit free exchange of viral populations between plasma and CSF, particularly in those infected with highly pathogenic subtype D virus. Although HIV-1 populations in CSF and plasma during CM have not been previously studied in subtype D infections, we expected viral variants to be equilibrated between compartments. Unexpectedly, we found evidence of compartmentalization between the CSF and plasma viral populations before ART initiation in 22% (4/18) participants including those infected with subtype D.

HIV-1 RNA in CSF is a fundamental feature of infection and viral levels, reflecting interactions between host and viral factors. Compartmentalization of HIV-1 virus between CSF and plasma has been reported in some PWH, with or without co-infection [8,10,13,14,16,17,22–24,29]. To investigate mechanisms driving viral persistence across compartments, we compared HIV-1 populations in CSF and blood among PWH with CSF viral RNA levels either higher or lower than in plasma. Consistent with Adewumi et al. [24] and Sojane et al. [29], we found no associations between HIV-1 compartmentalization and clinical outcomes (death, IRIS), viral characteristics (subtype, plasma of CSF HIV-1 RNA levels, genetic diversity, *env* tropism), CM characteristics (CrAg levels, CSF WBC counts), or most immune parameters (CD4 proportion, TH1, TH2, TH17, myeloid cytokines) [24,29]. However, several cytokines (IL-2, IL-7, IL-10, GMCSF, MCP-1, IL-8) were significantly elevated in CSF compared to plasma, and CSF IL-2 levels correlated with viral compartmentalization. Elevated IL-2 has also been reported in cases of *Mycobacterium tuberculosis* co-infections where compartmentalized HIV-1 variants were detected in pleural fluid and blood [44]. Increased CSF cellularity was also associated with higher CSF viral loads, consistent with prior cohorts [6,15,45], supporting the interplay of immune activation and viral replication [45]. Alternatively, we cannot rule out that expansion of a subset of HIV-1 variants may also give the appearance of evolution. Whether compartmentalized viral populations contribute to cytokine elevation, or whether cytokine increases solely reflect downstream inflammation, remains unclear. These findings highlight the need for further investigations of host-virus interactions shaping HIV-1 population structure across anatomical compartment.

Analysis of *env* tropism identified both CXCR4- and CCR5-utilizing viruses in CSF and blood compartments. In some participants, compartmentalized viral populations were dominated by either CXCR4- or CCR5-tropic variants, suggesting that specific clinical conditions may favor expansion of one population over the other. Overall, the inflammatory environment within the CSF appears to facilitate viral replication and the emergence of distinct CSF viral populations. We propose that these populations arise when HIV-1 infected cells traffic across the BBB into the CNS during active infection [15], evidenced by the similarity between CNS and plasma viral DNA. Once in the CNS, local inflammation may drive molecular evolution to produce distinct CSF viral populations from these infected cells. Similar trafficking and compartmentalization mechanisms have been described in other comorbid conditions, including Hepatitis C coinfection and brain injury [11,45]. Consistent with this hypothesis, several PWH with high levels (>10^5^ copies/ml) of compartmentalized virus had CSF populations composed of macrophage-tropic HIV-1, likely reflecting viral evolution within the microglial compartment of the CNS. Because macrophage-tropic HIV-1 is typically detected at low levels, the elevated levels we observed may reflect neuroinflammation during active CM, amplifying macrophage tropic viral replication.

Prior studies show that HIV-1 in the CNS predominantly infects CCR5-utilizing cells [46,47]. However, in our study, CXCR4-utilizing HIV-1 populations were frequently detected in CSF and were concordant with plasma populations, suggesting shifts in coreceptor use that may occur with lymphopenia [48]. CCR5-utilization was associated with CSF pleocytosis, a marker linked to improved prognosis in CM [49,50], indicating the immune system may not be as severely impaired. A significant proportion of our participants also had subtype D HIV-1 infections, which is known to exhibit higher frequencies of CXCR4-tropic variants. However, our coreceptor predictions were consistent across geno2pheno and other subtype D prediction algorithms [34,51,52].

HIV-1 population diversity in both CSF and plasma during CM was substantial, although CSF contained fewer unique HIV-1 variants than plasma. Some of these variants may be macrophage-tropic, potentially contributing to CM-associated neuroinflammation [8,10,53]. One participant (P6) had CSF and plasma specimens available after therapy in the setting of CM relapse and increase in HIV-1 viremia, providing insight into the viral dynamics of this individual’s HIV-1 populations post-ART. New viral lineages present in the CSF that were not detected pre-ART, could reflect limited sampling depth [4] or expansion following seeding of a CNS reservoir and persistent replication in this compartment [8,10,53,54]. This observation suggests the possibility that during CM, productively infected cells migrate across plasma and CSF compartments, leading to the emergence of distinct HIV-1 populations following ART interruption. Further studies of rebound viremia following CM therapy may clarify whether HIV-1 populations persist and expand during ART [10].

Multiple infections may influence apparent viral compartmentalization. In our studies, we identified one individual with two distinct subtypes (subtypes A1 and C), as a result of dual infection (P18). Although subtype C was exclusively detected in plasma, there was insufficient sampling to deduce anatomic compartmentalization (N = 4 sequences) of this subtype. In several other participants, phylogenetic trees had well-supported divergent branches within the same subtype, suggesting multiple infection events. However, the presence of polymorphisms, insertions and deletions that were in linkage equilibrium across these large branches indicate frequent recombination among viral lineages, complicating our interpretations of compartmentalization. Therefore, larger longitudinal datasets are necessary to clarify the role of multiple infections on compartmentalization.

Our study has several limitations. Temporal viral dynamics and viral reservoir formation could not be determined because the majority of our pre-ART analyses represented a single timepoint. Interpretation of post-ART analyses for P6 is limited because CSF and plasma samples were collected at different, unpaired timepoints. Compartmentalization may be under-represented because some participants had too few unique CSF *env* sequences to adequately power the analyses. We also recognize that increased viral diversity in plasma may not sufficiently represent CSF populations [4] nor the potential impact of pleocytosis. Finally, as an exploratory study, we did not adjust for multiple comparisons to avoid missing potentially meaningful associations given the rarity of these samples [55].

In summary, we identified compartmentalized HIV-1 populations between CSF and plasma in a subset of PWH during CM. This compartmentalization likely reflects the distinct CSF inflammatory milieu characterized in part by elevated IL-2, potentially supporting macrophage-tropic viral populations. These findings suggest that opportunistic infections and neuroinflammation can shape HIV-1 replication dynamics within the CNS, reinforcing the importance of earlier diagnosis and treatment. Importantly, clinical, immunologic and virological events occurring upon ART initiation may influence viral populations that seed long-lived HIV-1 reservoirs. Prior work [54,56] demonstrated that viruses present at ART initiation often re-emerge during treatment interruption. Whether similar rebound viruses originate from CNS reservoirs remains unclear, but defining these mechanisms will be critical for the development of new remission strategies.

## Materials and methods

### Sex as a biological variable

The COAT (NCT01075152) and ASTRO-CM (NCT01802385) studies enrolled male and female participants that met study criteria. Findings are reported for both sexes.

### Study participant cohort

The Cryptococcal Optimal ART Timing (COAT) was a randomized clinical trial in South Africa and Uganda evaluating early versus deferred antiretroviral therapy in PWH and cryptococcal meningitis that began enrollment on November 22, 2010 and ended enrollment on April 27, 2012. The Adjunctive Sertraline for the Treatment of HIV-Associated Cryptococcal Meningitis (ASTRO-CM) was a randomized clinical trial evaluating the use of adjunctive sertraline in cryptococcal meningitis that began enrollment March 9, 2015 and ended enrollment May 29, 2017. Pre-ART CSF and plasma were evaluated in 39 study participants from the COAT and ASTRO-CM trials.

Single genome sequence data from viral RNA were generated in a subset of 18 study participants (S1 Table and S2 Table) from pre-ART timepoints. Cell-associated DNA-derived single genome sequences were generated from paired PBMC and CSF cytospin cells which were also available from the pre-ART timepoints in 5 of those 18 participants (S2 Table). Neurocognitive outcomes were calculated using a quantitative neurocognitive performance z-score as previously described [31].

### CSF analysis

HIV-1 RNA levels in plasma and CSF were measured with the Abbott (Abbott Park, IL) Alinity m Real-Time PCR analyzer (LOD 20 copies/mL) according to the manufacturer’s instructions. CSF cell enumeration and chemistry analyses were performed on site in laboratories participating in external quality assurance testing as formerly described [57]. CSF cytokines and chemokines (G-CSF, GM-CSF, IFN-γ, TNFα, IL-1B, IL-2, IL-4, IL-5, IL-6, IL-7, IL-8, IL-10, IL-12, IL-13, IL-17, MCP, MIP1β) were measured from cryopreserved CSF samples (available on 17 of the 18 study participants analyzed) at the University of Minnesota using Luminex magnetic bead technology as formerly described [57]. Plasma cytokines and chemokines were available for six participants and paired analyses were performed on these CSF and plasma biomarkers.

### Single genome sequencing

Single genome sequences encoding full-length HIV-1 envelope (*env*) were obtained from viral populations in plasma and cells [4,58] (See S5 Table for sequencing primers). Sequence analyses were performed with Version X of the Molecular Evolutionary Genetic Analysis Software (MEGA X; https://www.megasoftware.net). APOBEC3G/F hypermutated sequences were identified with Hypermut 4.0 (https://www.hiv.lanl.gov/content/sequence/HYPERMUT/hypermut.html) and excluded from sequence analyses.

### Coreceptor utilization and macrophage tropism

CCR5 co-receptor utilization of *env* sequences was determined with Geno2pheno [coreceptor] using a series of false positive cutoff of <2.6 (see S4 Table; https://coreceptor.geno2pheno.org/ [59]). To specifically investigate macrophage tropism, selected *env* sequences were evaluated for utilization of low levels of CD4 using the affinofile assay [37,60]. Briefly, *env* sequences were synthesized (GenScript), then cloned into pNL4–3 based vectors, transfected in HEK293 cells, viral infectivity measured in affinofile cells as previously described [37,60,61].

### Population genetics analyses

HIV-1 subtypes were determined for all single genome sequences with the REGA HIV-1 Automated Subtyping Tool [62]. Diversity was measured by average pairwise distances (APD) of HIV-1 RNA and DNA populations and Hill’s diversity was also used for HIV-1 RNA populations. Neighbor-joining phylogenetic trees were constructed with MEGA X, aligned and trimmed to HXB2 *env* (Accession K03455.1) which was used as the outgroup for phylogenetic reconstructions. Identical sequences were detected with ElimDupes tool provided by the Los Alamos National Laboratory http://www.hiv.lanl.gov/ [63]. We used non-parametric [35] and parametric tests to determine the presence of panmixia and structural compartmentalization between HIV-1 populations respectively. Non-parametric analysis is an adaptation of the Hudson geographic subdivision test to detect differences in population composition in samples from plasma and CSF. By comparing the average number of pairwise differences between sequences within each population to a theoretical population obtained by randomly shuffling the sequences, this test estimates the probability that the two samples of viral sequences are drawn from a single population. We also performed gene flow analyses using phylogenetic approach of Slatkin and Maddison. This test employs a permutation approach to estimate whether the minimum number of migrations from a maximum parsimony tree is lower than that expected from a panmictic population (implemented using the Slatkin module in HyPhy vision (http://vision.hyphy.org/Slatkin). Analyses were performed with and without identical sequences in each population to determine if identical sequences and/or changes in genetic composition of HIV-1 populations accounted for differences between populations.

### Statistical analysis

Baseline clinical characteristics of the 39 study participants were summarized with frequencies or medians and interquartile ranges as appropriate. Demographic, CSF biomarker data were compared by CSF and plasma compartment viral load with the chi-square and Mann-Whitney tests for nominal and ordinal data respectively. Neuro-cognitive testing by CSF:plasma ratios and CSF pleocytosis and CCR5 co-receptor utilization were compared using Mann-Whitney testing. Paired analysis of viral loads, average pairwise difference, CSF and plasma biomarkers were performed using two-sided Wilcoxon signed rank test. Comparisons between compartmentalization or identical sequences and clinical characteristics, CSF cytokines/chemokines, CSF profile were analyzed using chi-square and Mann-Whitney tests for nominal and ordinal data respectively. Given the small sample size and exploratory analysis, no corrections were performed for multiple comparisons [55]. Statistical analysis was done in R version 4.3.0 and figures were created with GraphPad Prism.

### Study approval

The COAT and ASTRO-CM trials were approved by site specific institutional review boards, at Mulago Hospital, Kampala, Mbarara Hospital, Mbarara, and GF Jooste Hospital in Cape Town South Africa for the COAT Trial and Mulago Hospital, Kampala, Mbarara Hospital, Mbarara, and University of Minnesota for the ASTRO-CM Trial. All participants provided written informed consent prior to participation.

## Supporting information

S1 Fig Neurocognitive scores. **A.** Neurocognitive scores did not differ by CSF and plasma viral load ratios (p = 0.077) **B.** CSF subtype (available on 10 participants, p = 0.15). Median and IQR are shown. Comparisons by two-sided Mann-Whitney test.
(TIF)

S2 Fig Phylogenetic reconstruction of HIV-1 env populations. Neighbor-joining tree of all HIV-1 *env* single genomes generated per study participant. Each study participant taxa are respectively color-coded as indicated in the key. HIV-1 subtypes indicated per participant. Bootstraps >99 shown.
(TIF)

S3 Fig Diversity of CSF versus plasma HIV-1 populations during cryptococcal meningitis. HIV-1 RNA diversity in CSF versus plasma with similar CSF and plasma average pairwise distance in five of eighteen subjects. Comparisons done by Wilcoxon signed rank test (p = 0.268).
(TIF)

S4 Fig Compartmentalization of HIV-1 DNA and RNA populations. Compartmentalization of HIV-1 populations in CSF, Plasma, CSF cells and PBMCs. Participants without compartmentalization between CSF and plasma compartments are depicted with blue squares and participants with compartmentalization between compartments are depicted with yellow squares. Compartmentalization analyses were conducted with both non-parametric and parametric approaches, with identical results [35,36].
(TIF)

S5 Fig HIV-1 Compartmentalization and CSF biomarkers. CSF cytokines in study participants with geographic subdivision and compartmentalization of CSF HIV-1 populations compared to those without compartmentalization. Median and IQR are shown. Comparisons done by Mann-Whitney tests, with significant differences in IL-2 (p = 0.007) ** 0.030 ≤ p ≤ 0.002. N = 17.
(TIF)

S6 Fig CSF and plasma cytokine analysis. Paired analysis performed using two-sided Wilcoxon signed rank tests with significant differences between plasma and CSF IL-2 (p = 0.03), IL-7 (p = 0.03), IL-10 (p = 0.03), GMCSF (p = 0.03), MCP1 (p = 0.03), MIP1b (p = 0.05), and IL-8 (p = 0.03). *0.030 ≤ p ≤ 0.050. N = 6. Median and IQR are shown.
(TIF)

S7 Fig Presence of CSF HIV-1 identical sequences and CSF biomarkers. CSF cytokines in study participants with identical HIV-1 env sequences detected in CSF compared to those without identical sequences; the total number of sequences studied were similar across all 17 patients, suggesting sensitivity to detect identical sequences was comparable in these datasets. Median and IQR are shown. Comparisons done by Mann-Whitney tests with significant differences in IFN (p = 0.039), TNF (p = 0.0495), IL-4 (p = 0.038), *0.030 ≤ p ≤ 0.050. N = 17.
(TIF)

S1 Table Baseline CSF biomarker levels at cryptococcal meningitis diagnosis. (XLSX)

S2 Table Baseline characteristics of eighteen participants. (XLSX)

S3 Table Single genomes generated from CSF and peripheral blood. (XLSX)

S4 Table Coreceptor utilization. Coreceptor utilization was predicted when false positive rate (FPR) <2.6 = CXCR4 utilization (Geno2pheno).
(XLSX)

S5 Table Summary of nucleotide primers used to generate HIV-1 *env* sequences. (XLSX)

S1 Appendix Phylogenetic trees of the eighteen study participants. Pre-ART Plasma HIV-1 RNA is represented by red squares. Pre-ART CSF HIV-1 RNA is represented by red circles. Pre-ART Plasma HIV-1 DNA is represented by black squares Pre-ART CSF HIV-1 DNA is represented by black circles. Open symbols are representative of predicted CCR5 coreceptor utilization and filled symbols are representative of predicted CXCR4 coreceptor utilization. Post ART populations are represented by blue branches. Red arrows indicate macrophage tropism. Stars are branches with bootstrap values >99.
(PDF)
