# Supplementary material for: HIV-1 compartmentalization in the central nervous system during cryptococcal meningitis: Analysis of viral, fungal, and host factors
Source: PLoS Pathog. 2026 Oct 15;22(10):e1014697. doi: 10.1371/journal.ppat.1014697 (PMC13671554; doi:10.1371/journal.ppat.1014697)

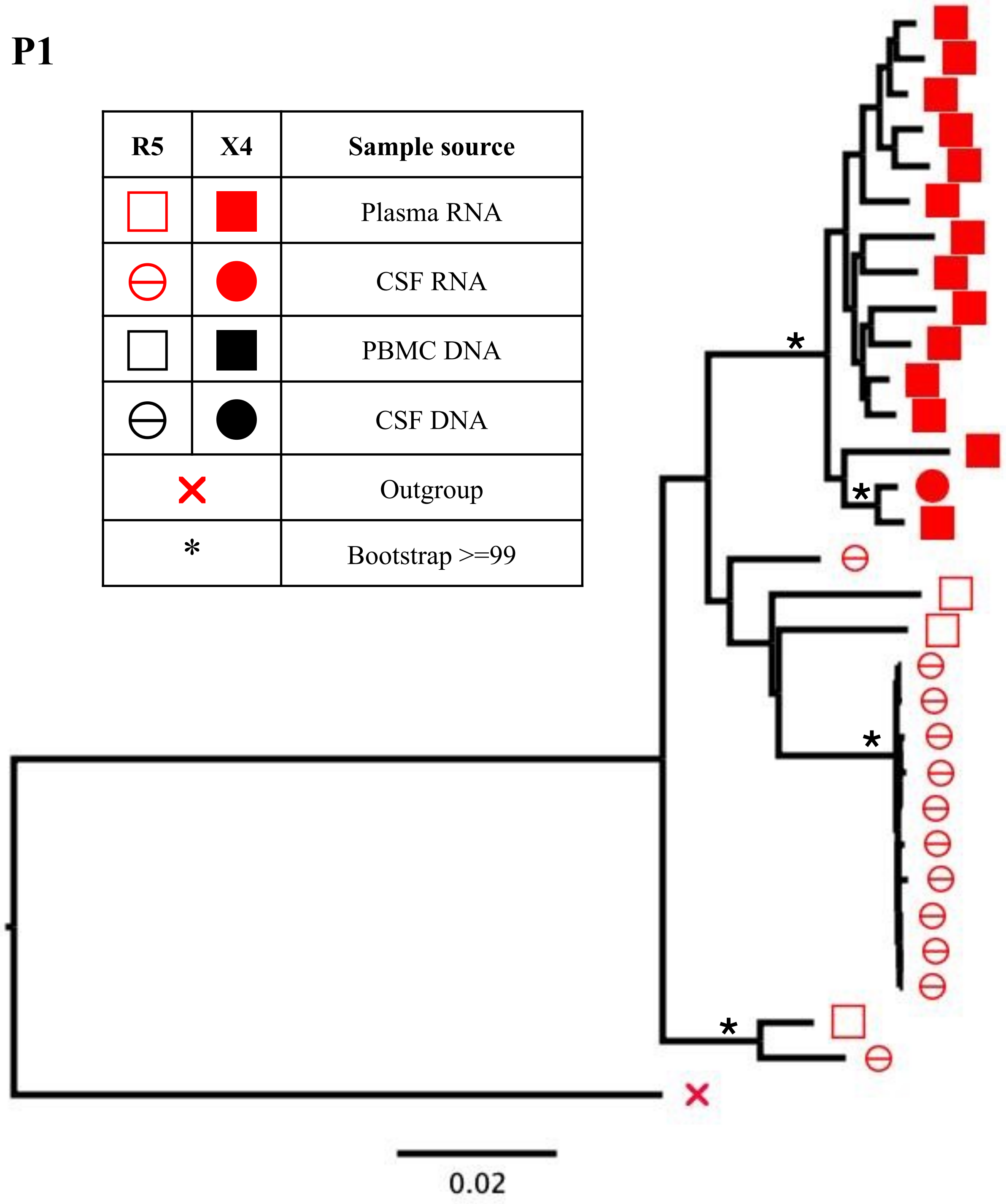

P1
R5
X4
Sample source
Plasma RNA
CSF RNA
PBMC DNA
CSF DNA
Outgroup
*
Bootstrap >=99
0.02

P2

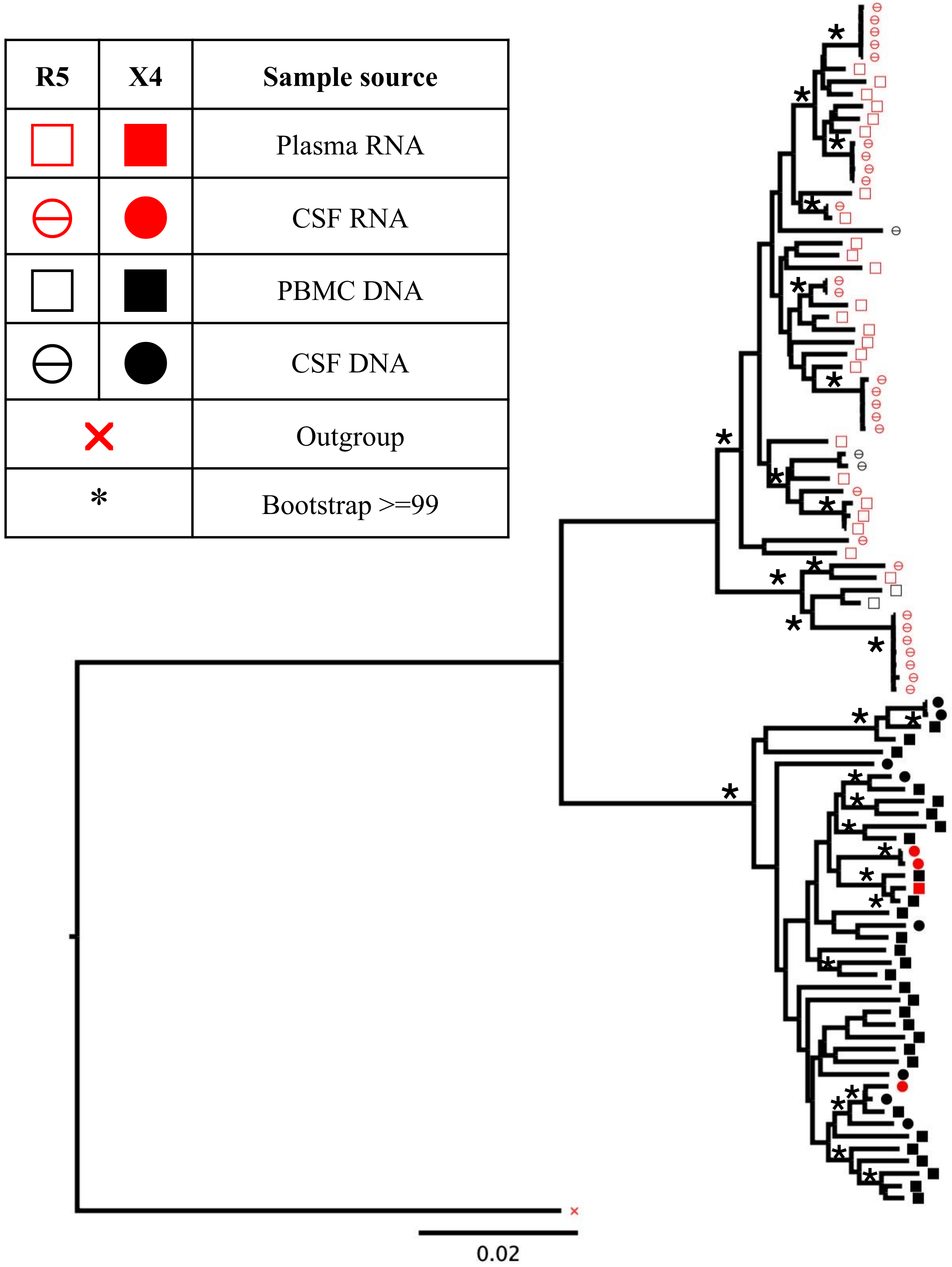
R5
X4
Sample source
Plasma RNA
CSF RNA
PBMC DNA
CSF DNA
Outgroup
*
Bootstrap >=99
0.02

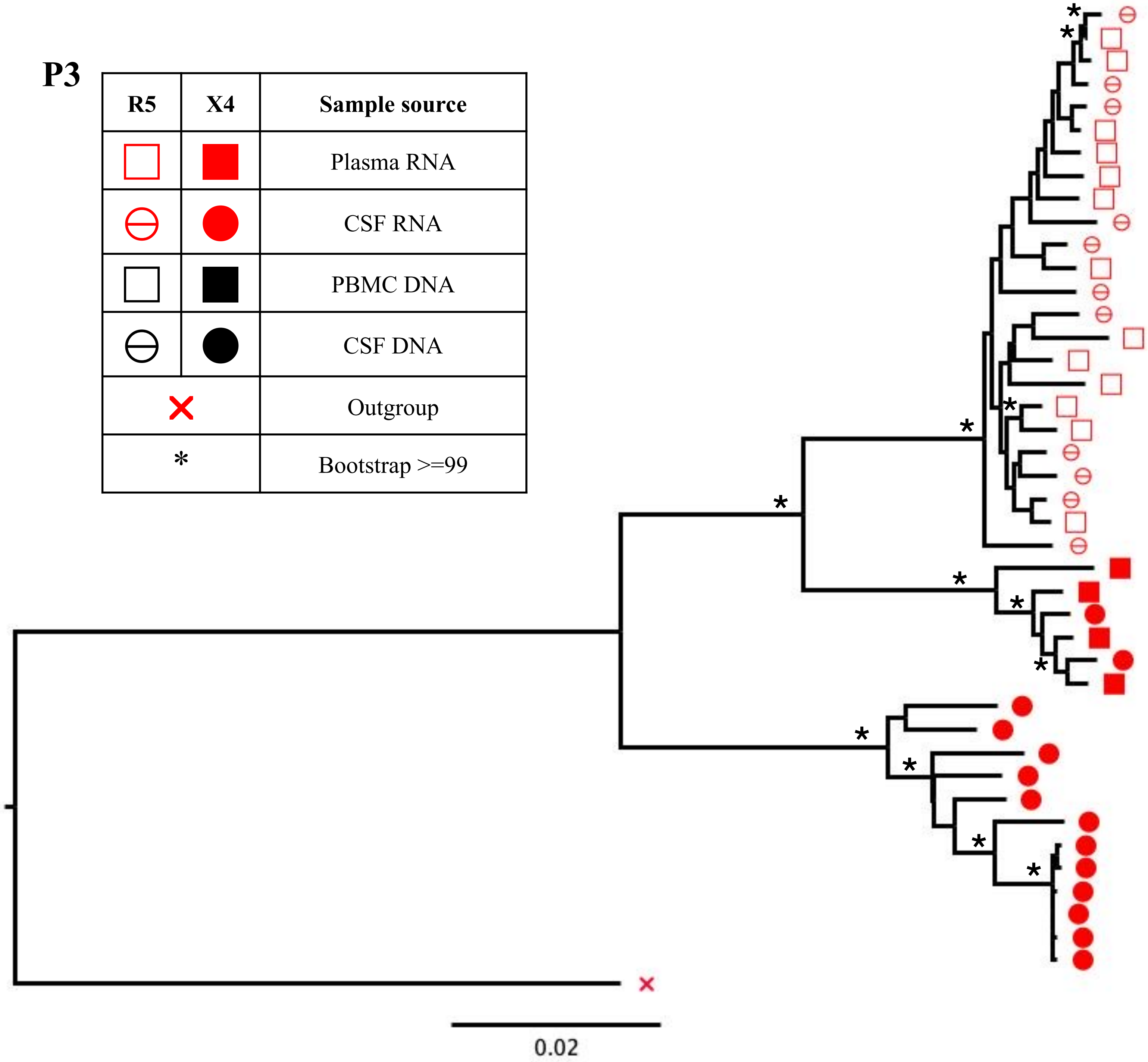
P3
R5
X4
Sample source
Plasma RNA
CSF RNA
PBMC DNA
CSF DNA
Outgroup
*
Bootstrap >=99
0.02

P4

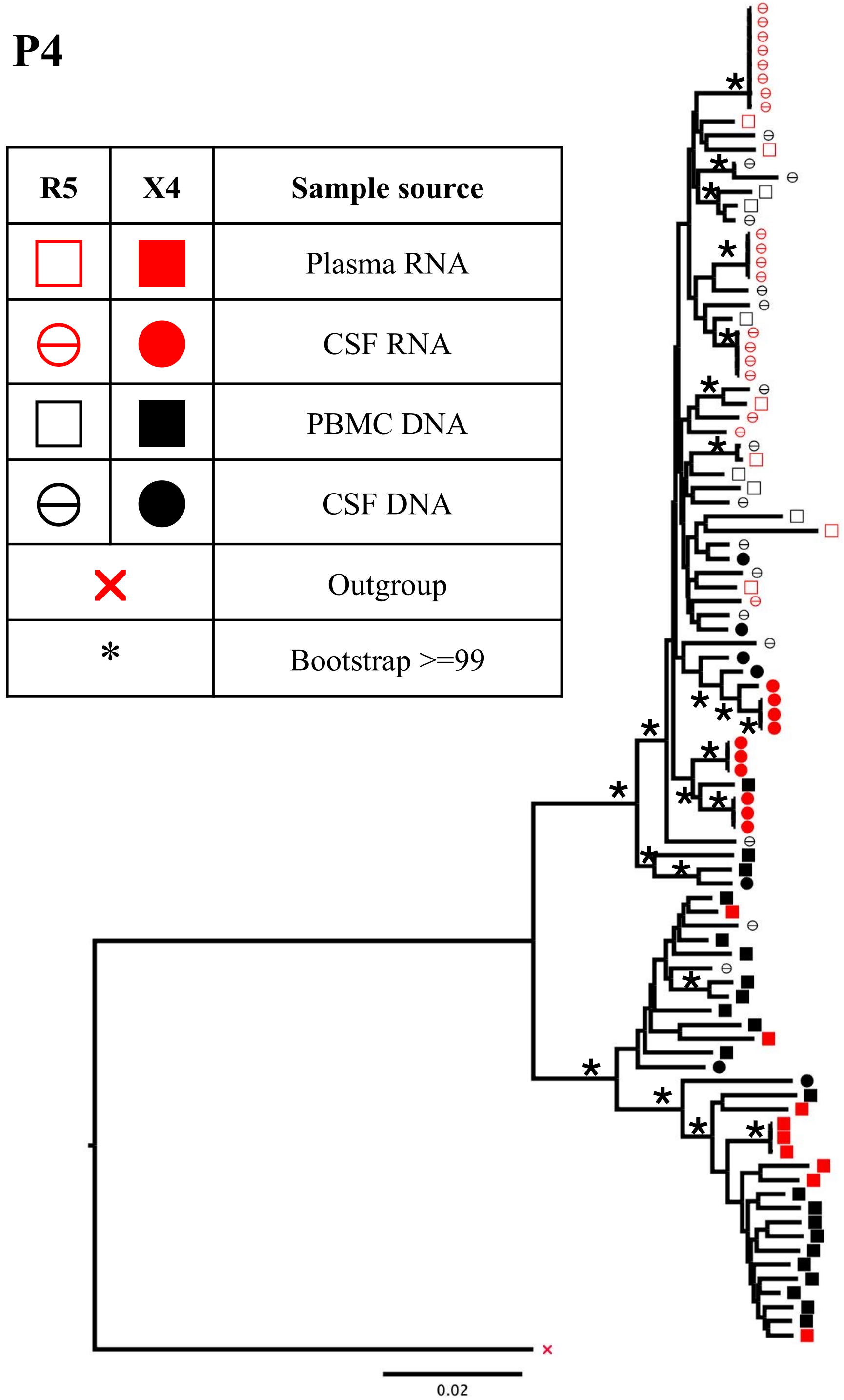
R5
X4
Sample source
Plasma RNA
CSF RNA
PBMC DNA
CSF DNA
Outgroup
Bootstrap >=99
0.02

P5

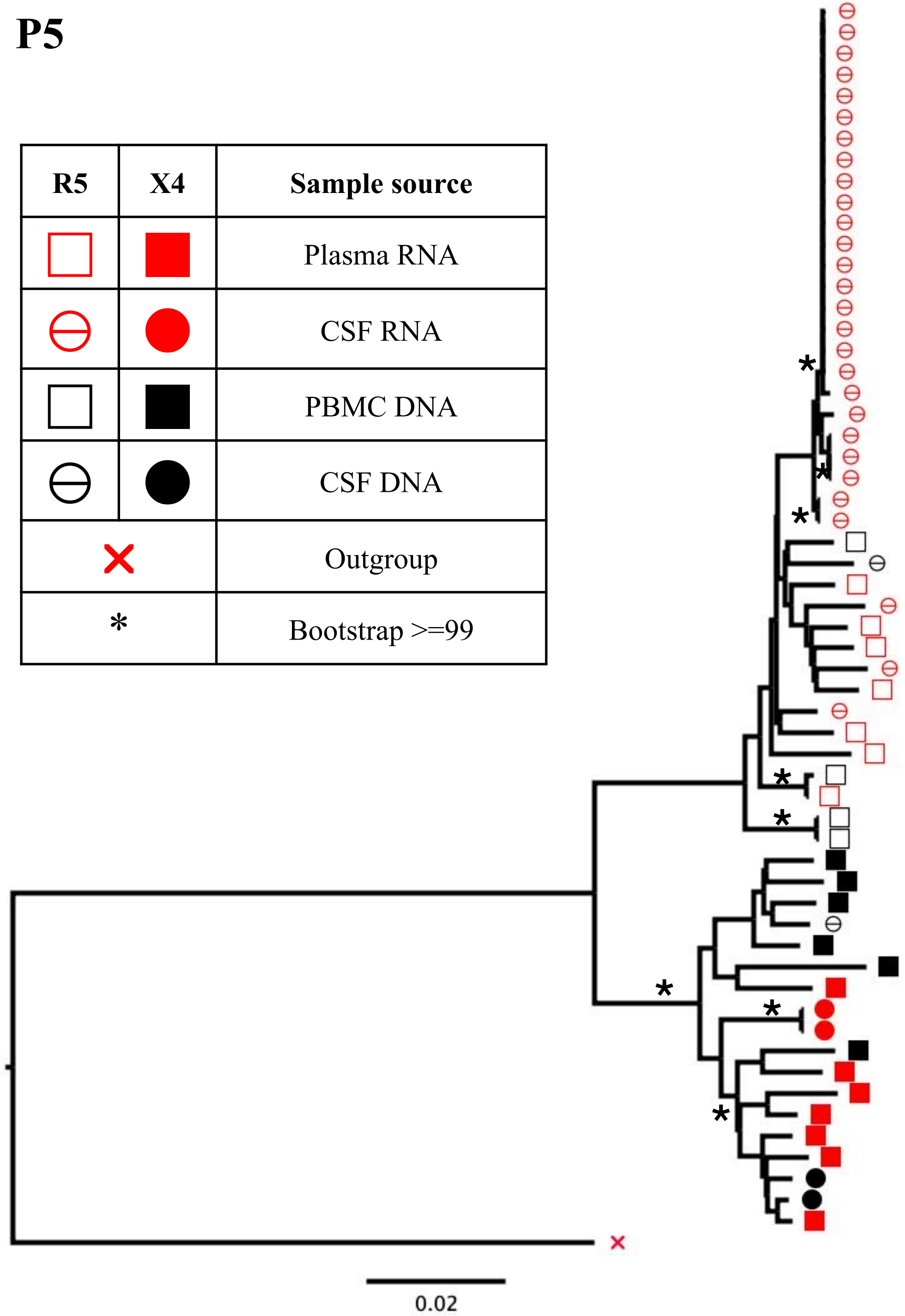
R5
X4
Sample source
Plasma RNA
CSF RNA
PBMC DNA
CSF DNA
Outgroup
Bootstrap >=99
0.02

P6

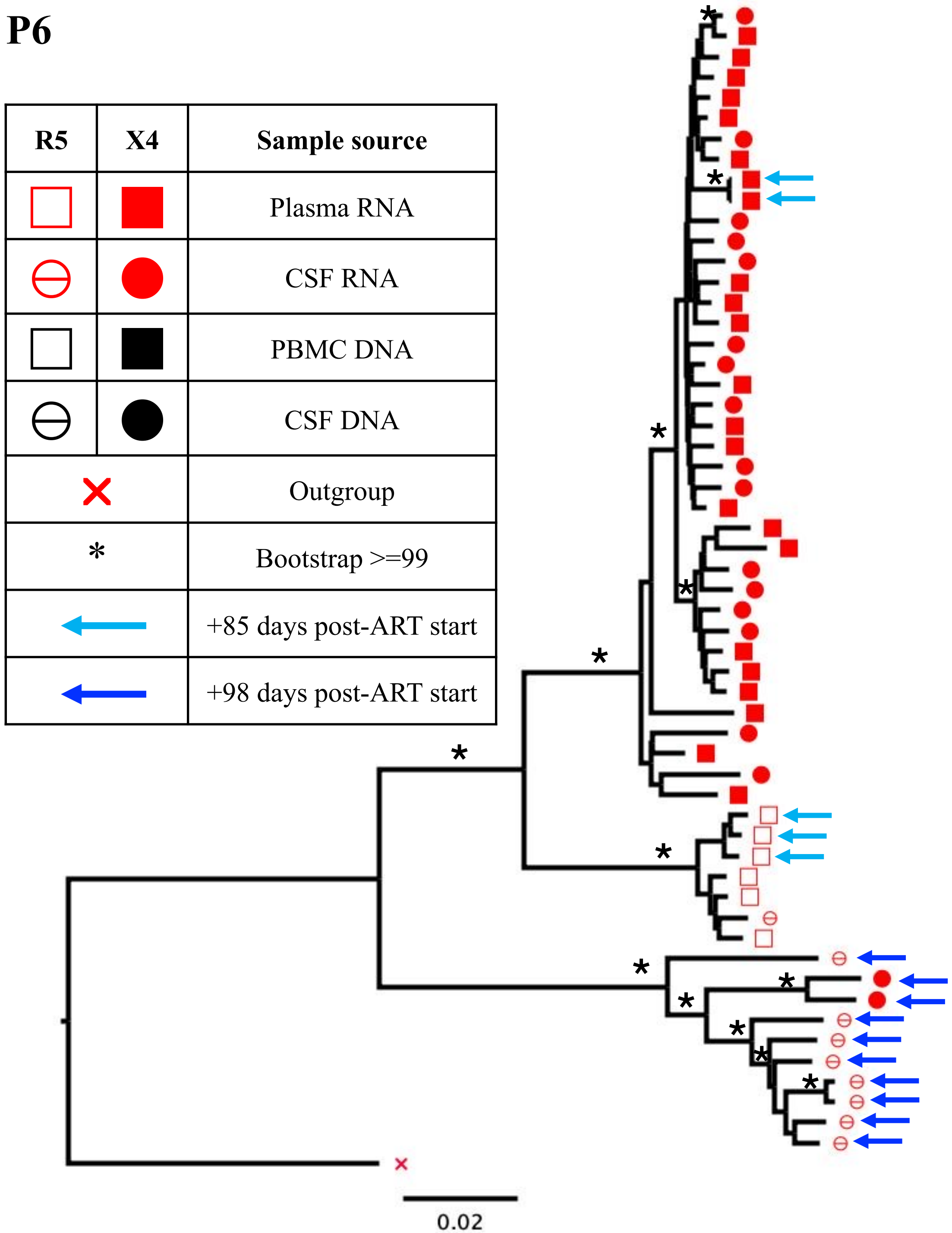
R5
X4
Sample source
Plasma RNA
CSF RNA
PBMC DNA
CSF DNA
Outgroup
Bootstrap >=99
+85 days post-ART start
+98 days post-ART start
0.02

P7

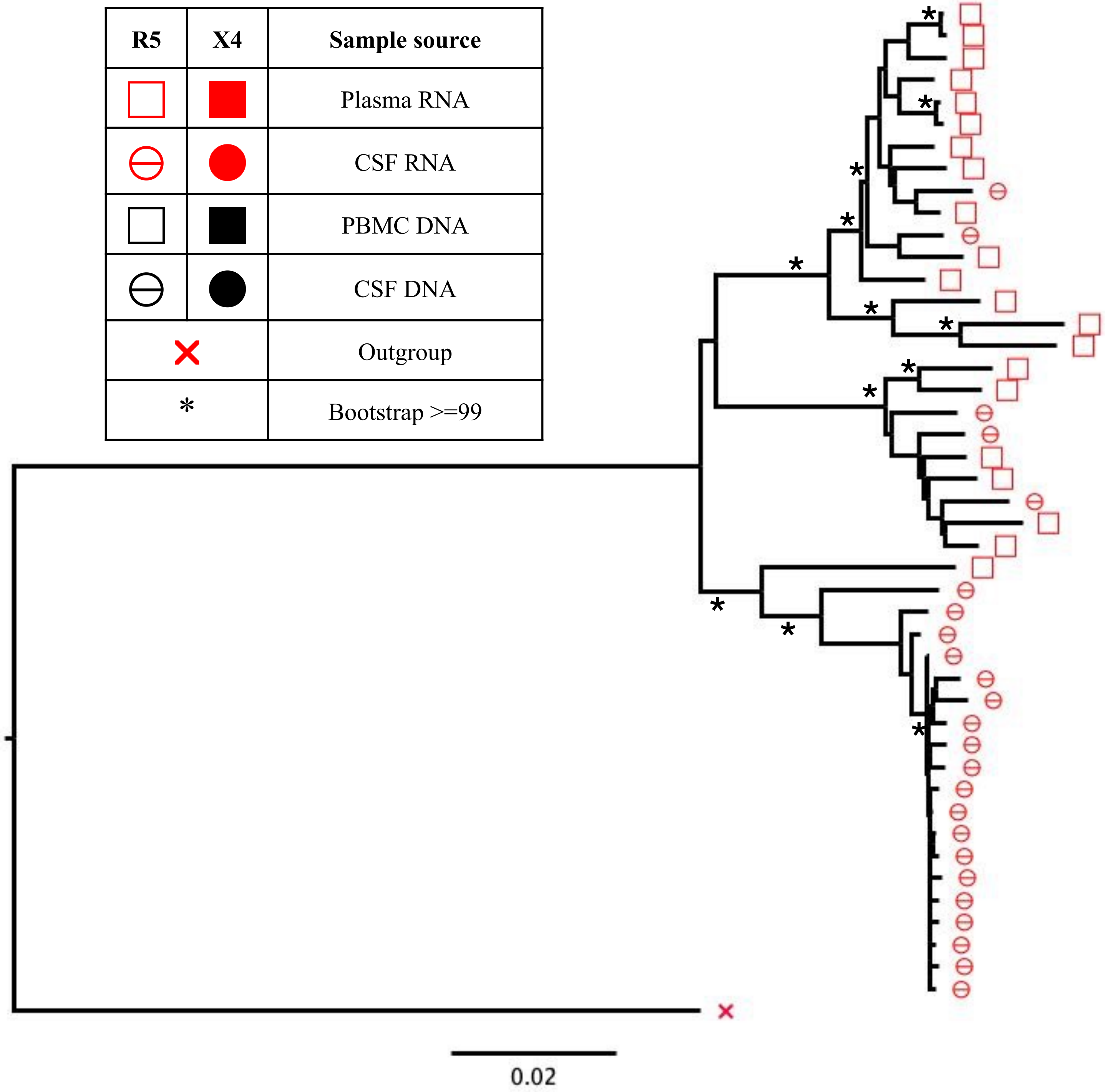
R5
X4
Sample source
Plasma RNA
CSF RNA
PBMC DNA
CSF DNA
Outgroup
*
Bootstrap >=99
0.02

P8

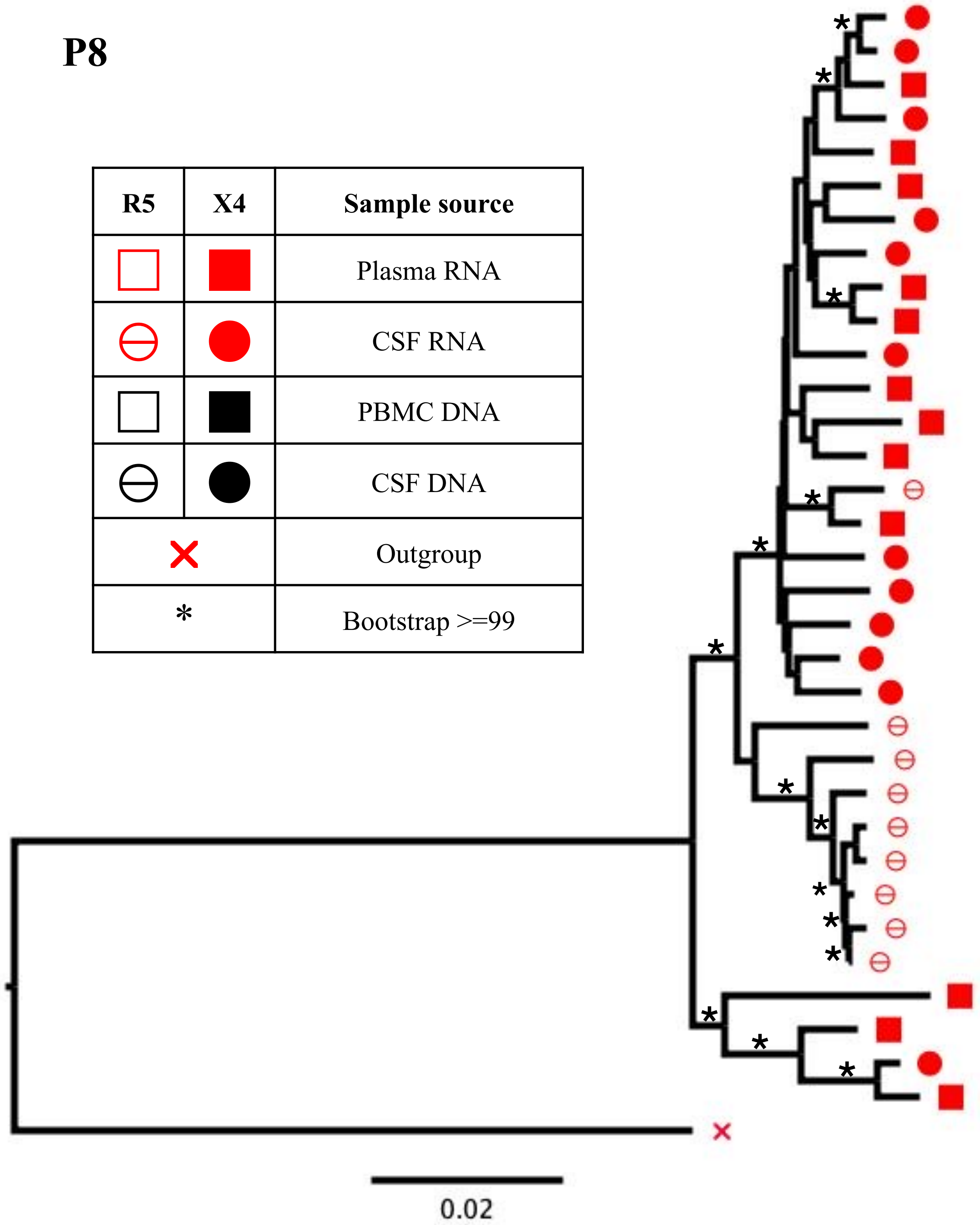

R5
X4
Sample source
Plasma RNA
CSF RNA
PBMC DNA
CSF DNA
Outgroup
*
Bootstrap >=99
0.02

P9

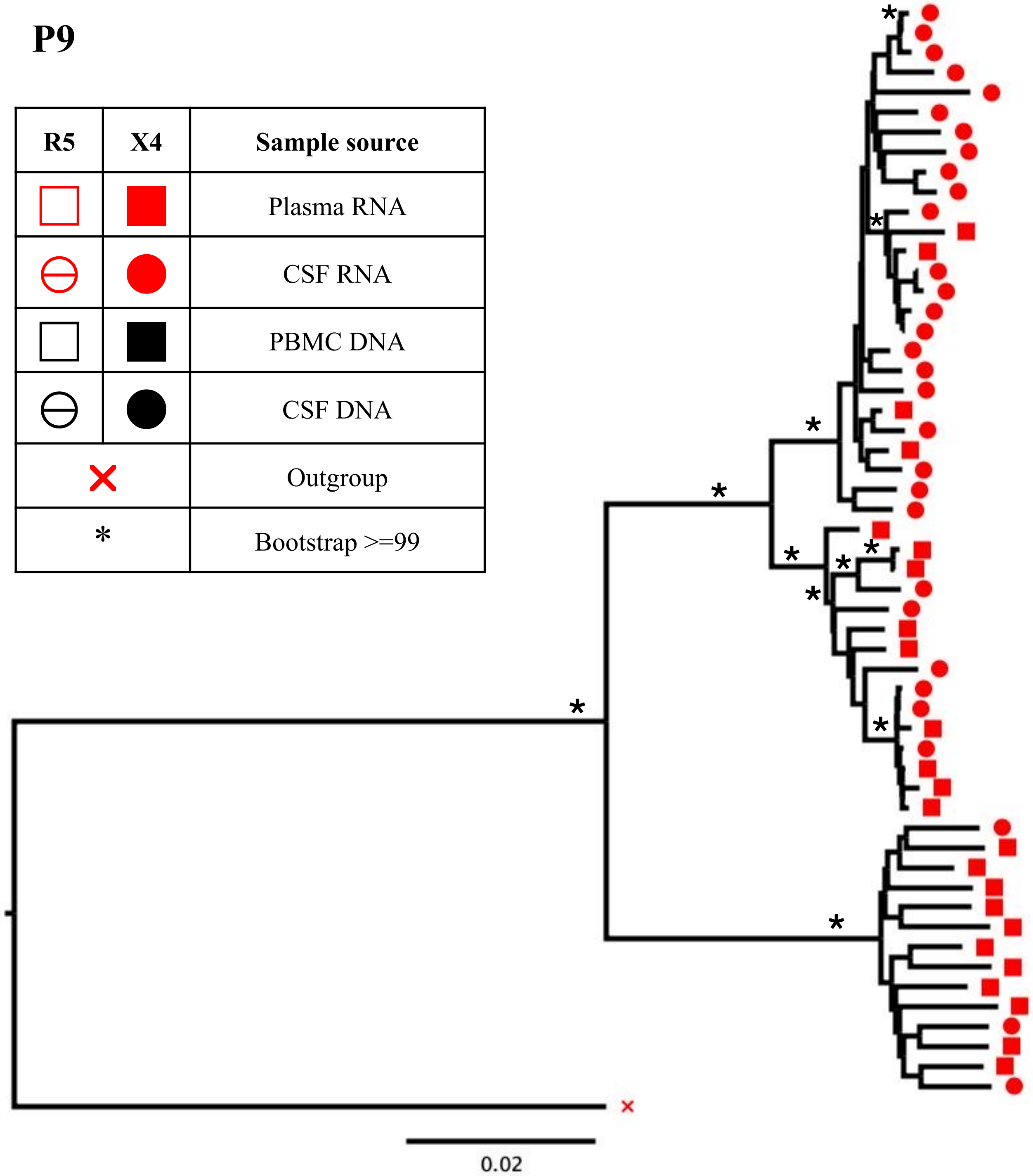
R5
X4
Sample source
Plasma RNA
CSF RNA
PBMC DNA
CSF DNA
Outgroup
*
Bootstrap >=99
0.02

P10

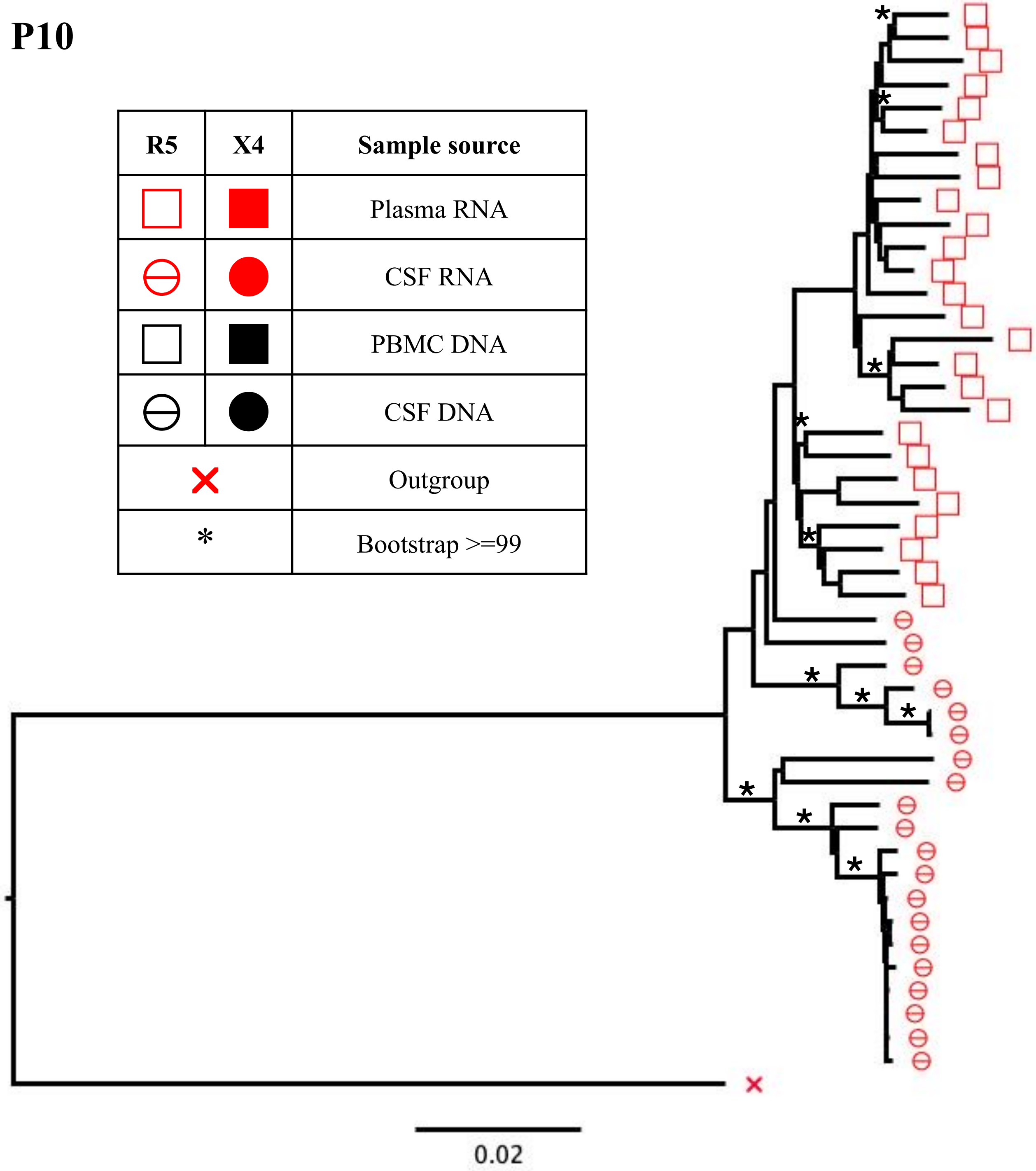
R5
X4
Sample source
Plasma RNA
CSF RNA
PBMC DNA
CSF DNA
Outgroup
*
Bootstrap >=99
0.02

P11

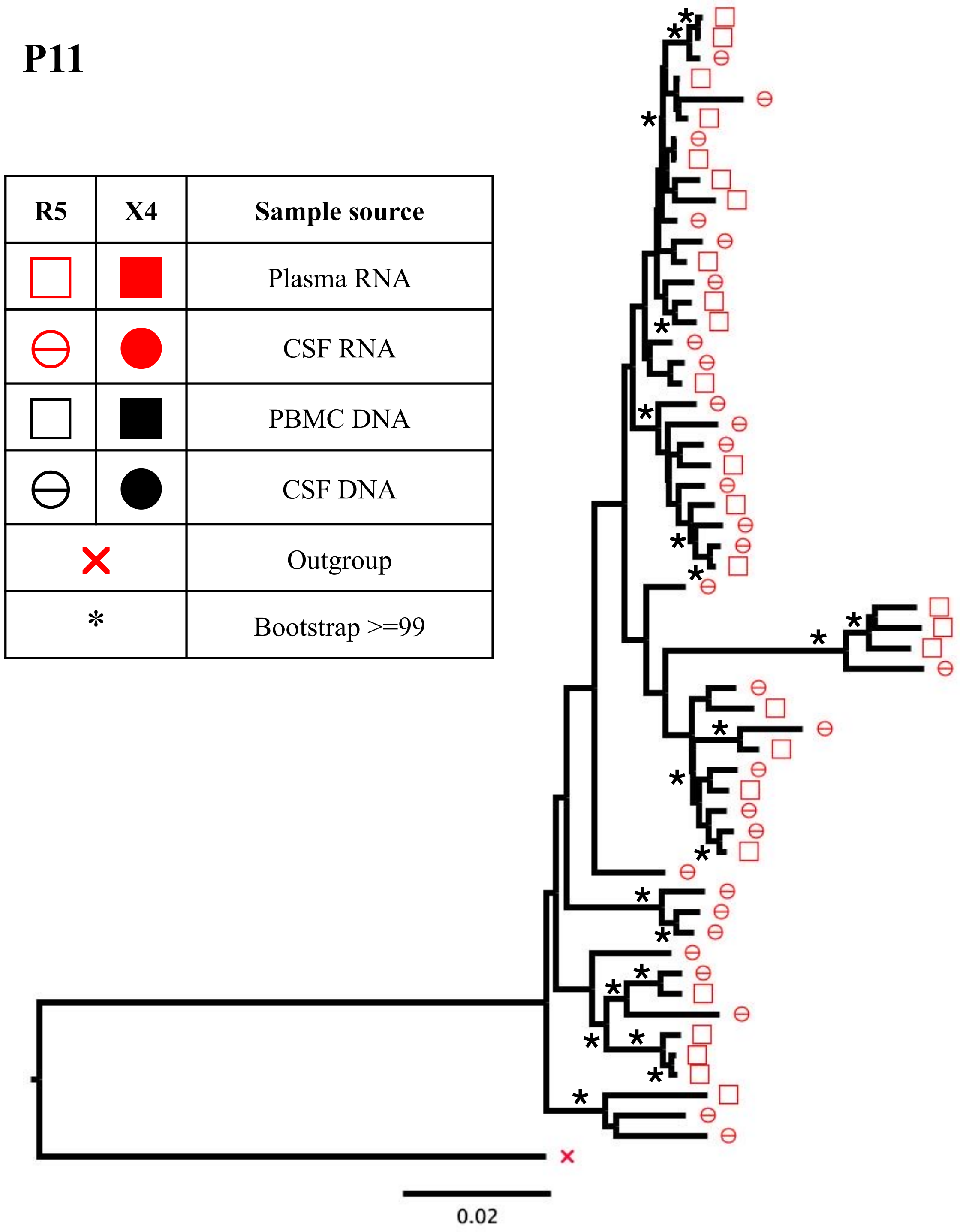
R5
X4
Sample source
Plasma RNA
CSF RNA
PBMC DNA
CSF DNA
Outgroup
*
Bootstrap >=99
0.02

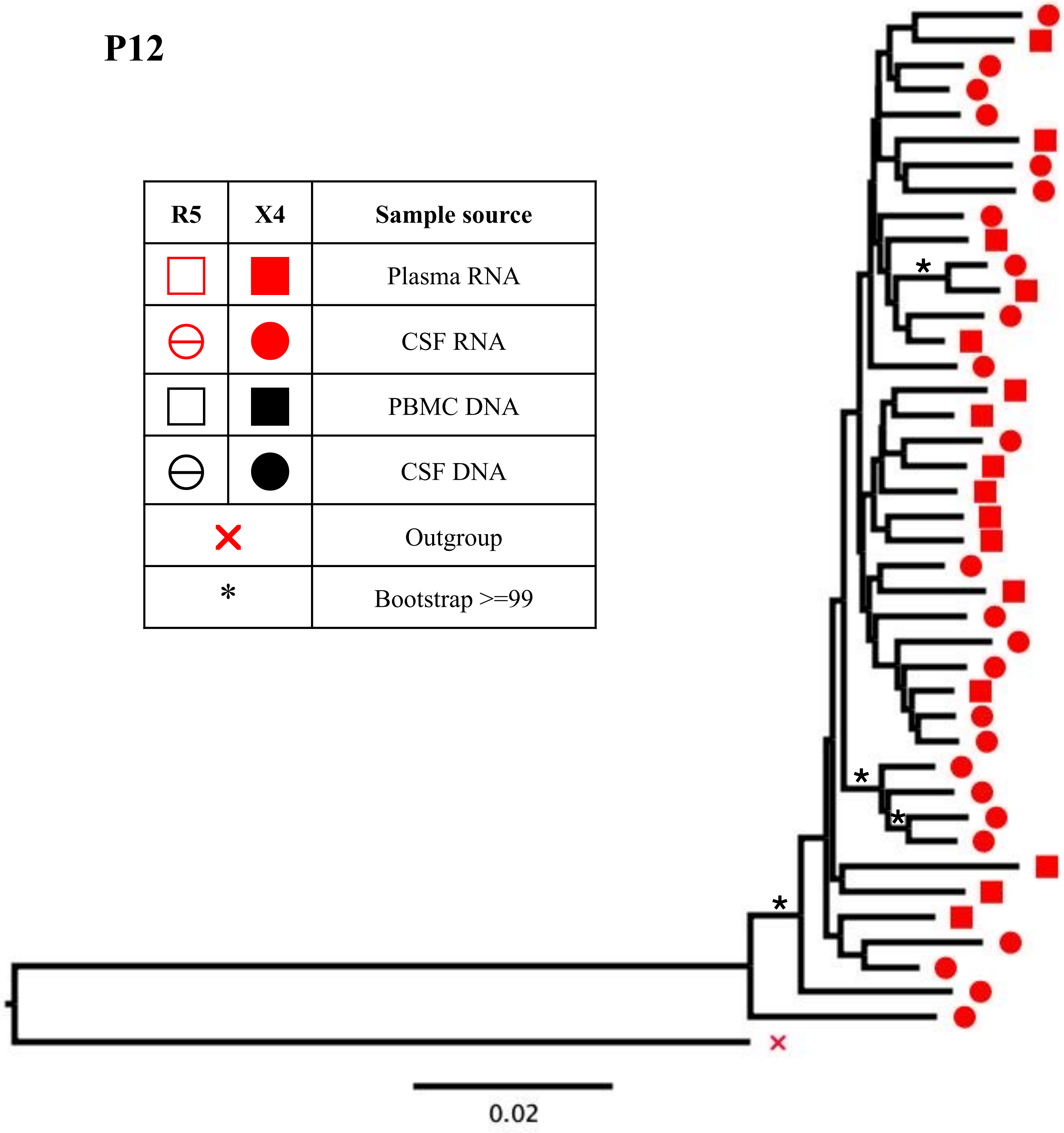

P12
R5
X4
Sample source
Plasma RNA
CSF RNA
PBMC DNA
CSF DNA
Outgroup
*
Bootstrap >=99
*
*
*
*
0.02

P13

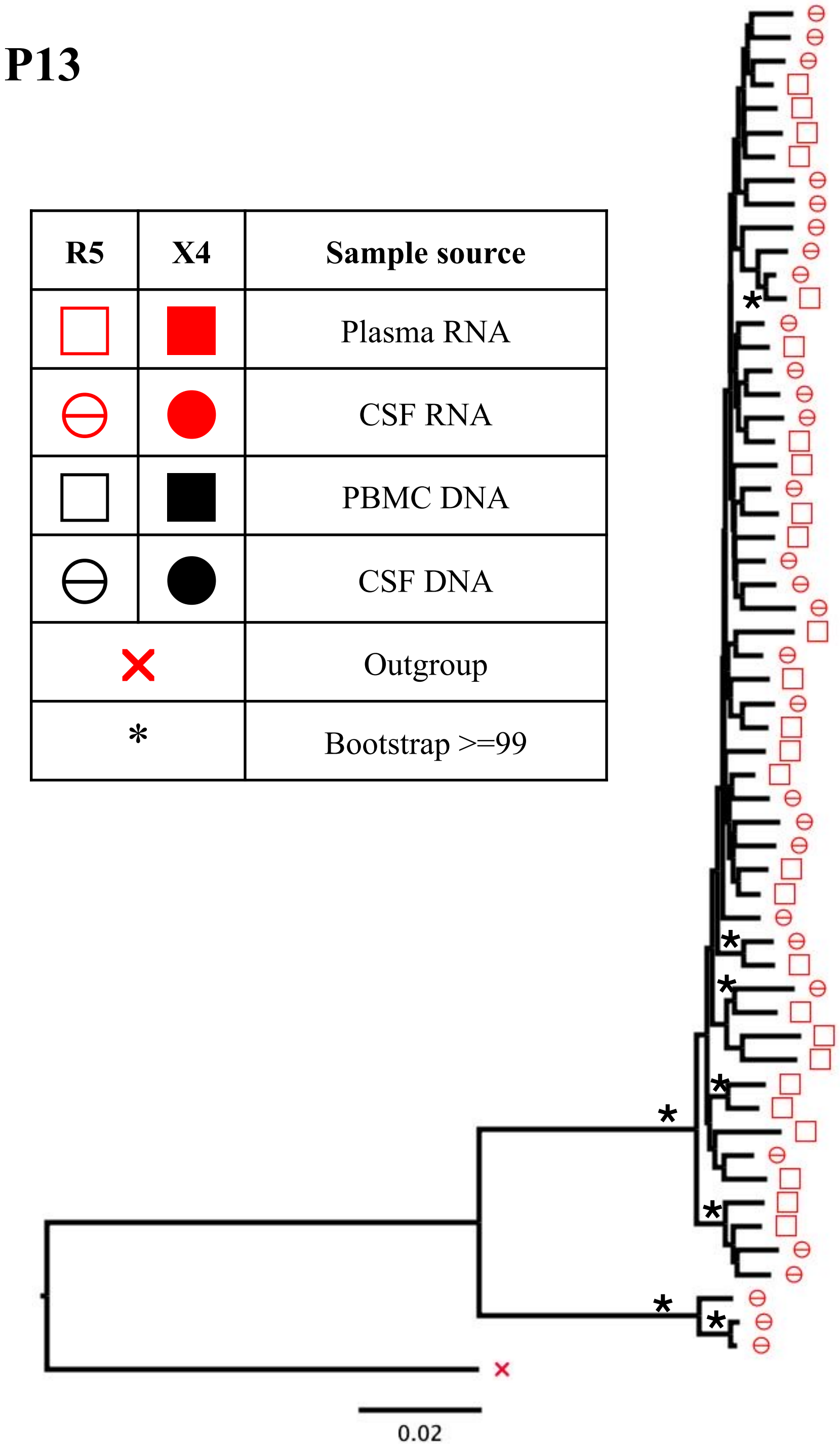

R5
X4
Sample source
Plasma RNA
CSF RNA
PBMC DNA
CSF DNA
Outgroup
*
Bootstrap >=99
0.02

P14

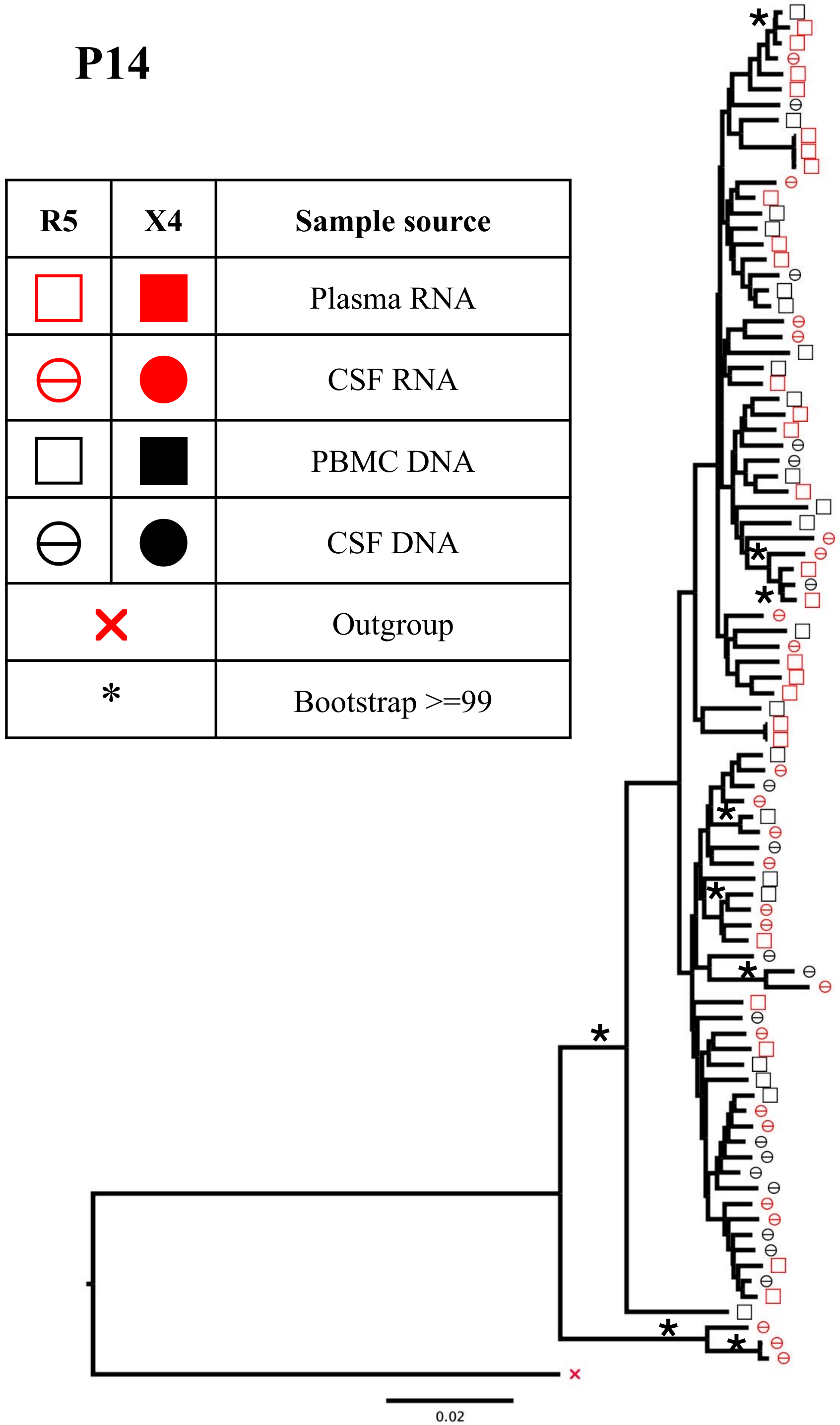
R5
X4
Sample source
Plasma RNA
CSF RNA
PBMC DNA
CSF DNA
Outgroup
*
Bootstrap >=99
0.02

P15

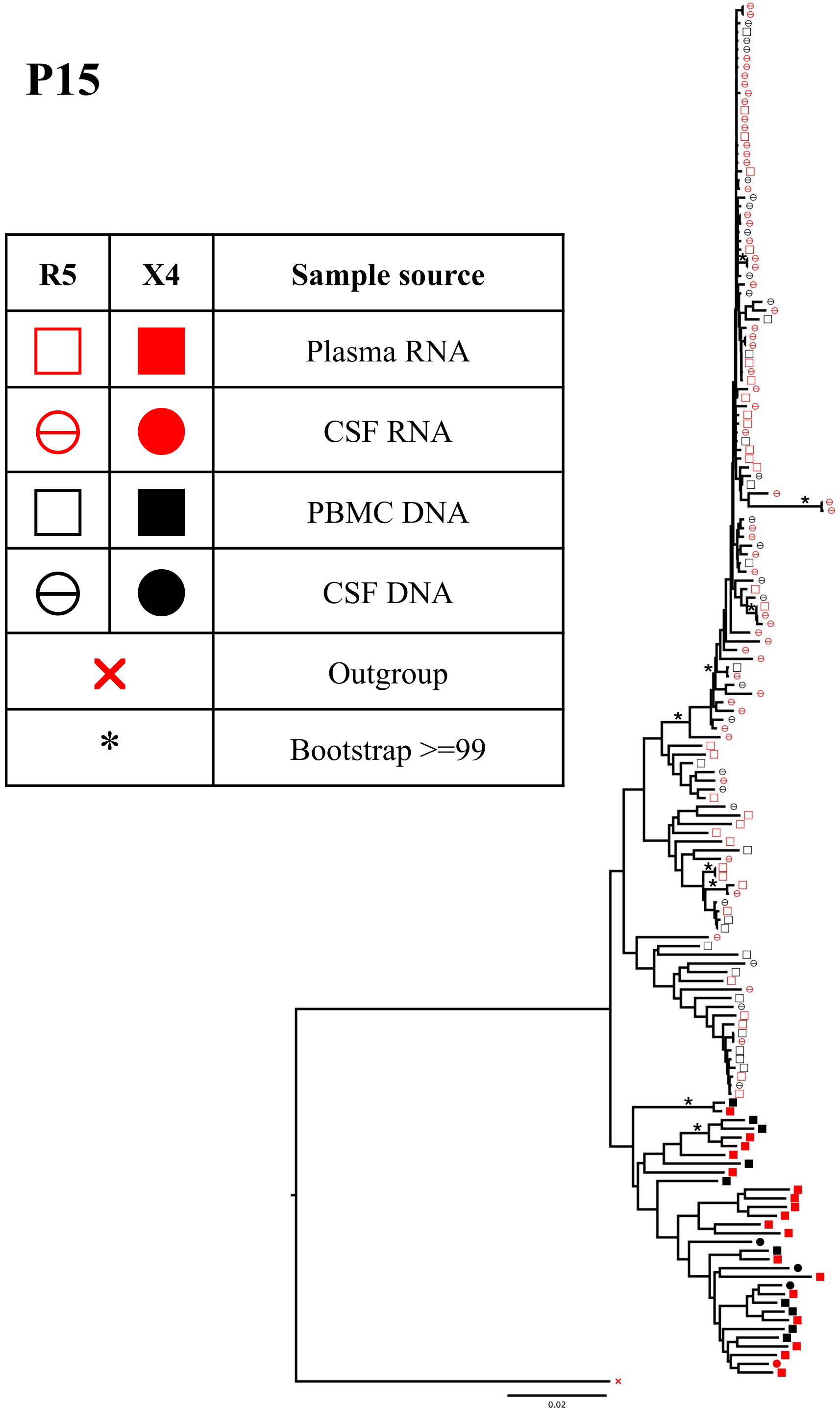
R5
X4
Sample source
Plasma RNA
CSF RNA
PBMC DNA
CSF DNA
Outgroup
*
Bootstrap >=99
0.02

P16

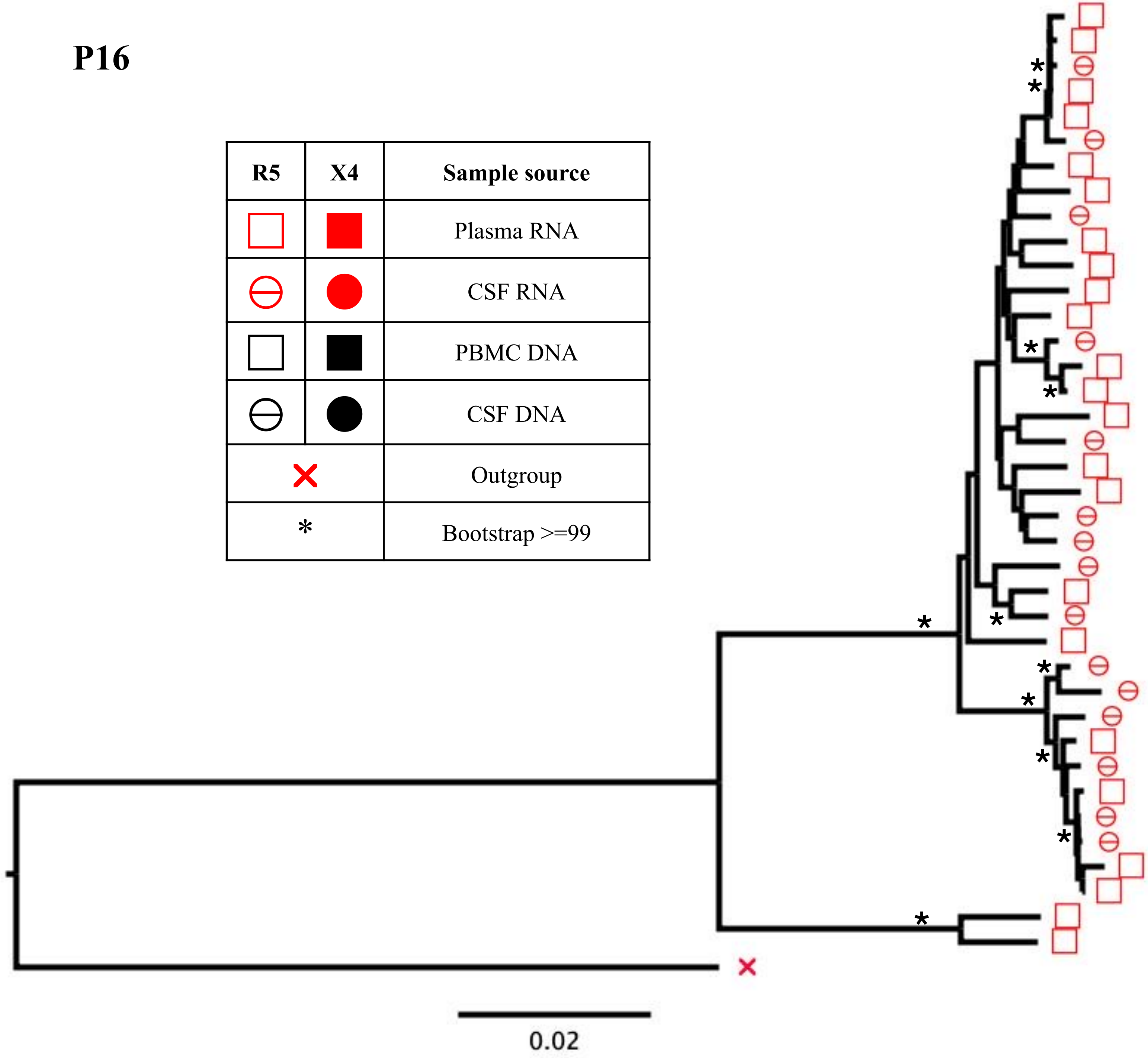
R5
X4
Sample source
Plasma RNA
CSF RNA
PBMC DNA
CSF DNA
Outgroup
*
Bootstrap >=99
0.02

P17

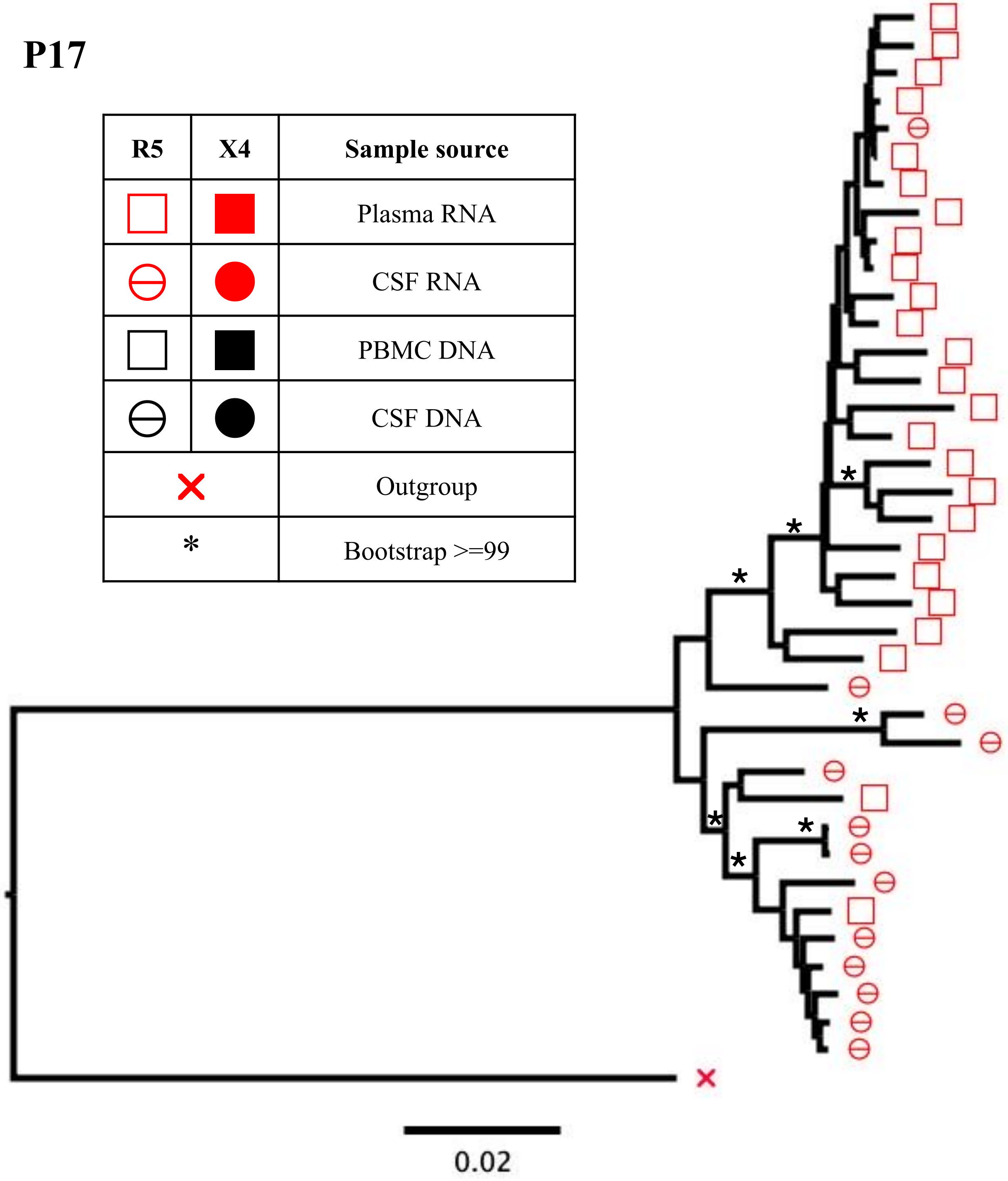

R5
X4
Sample source
Plasma RNA
CSF RNA
PBMC DNA
CSF DNA
Outgroup
*
Bootstrap >=99
0.02

P18

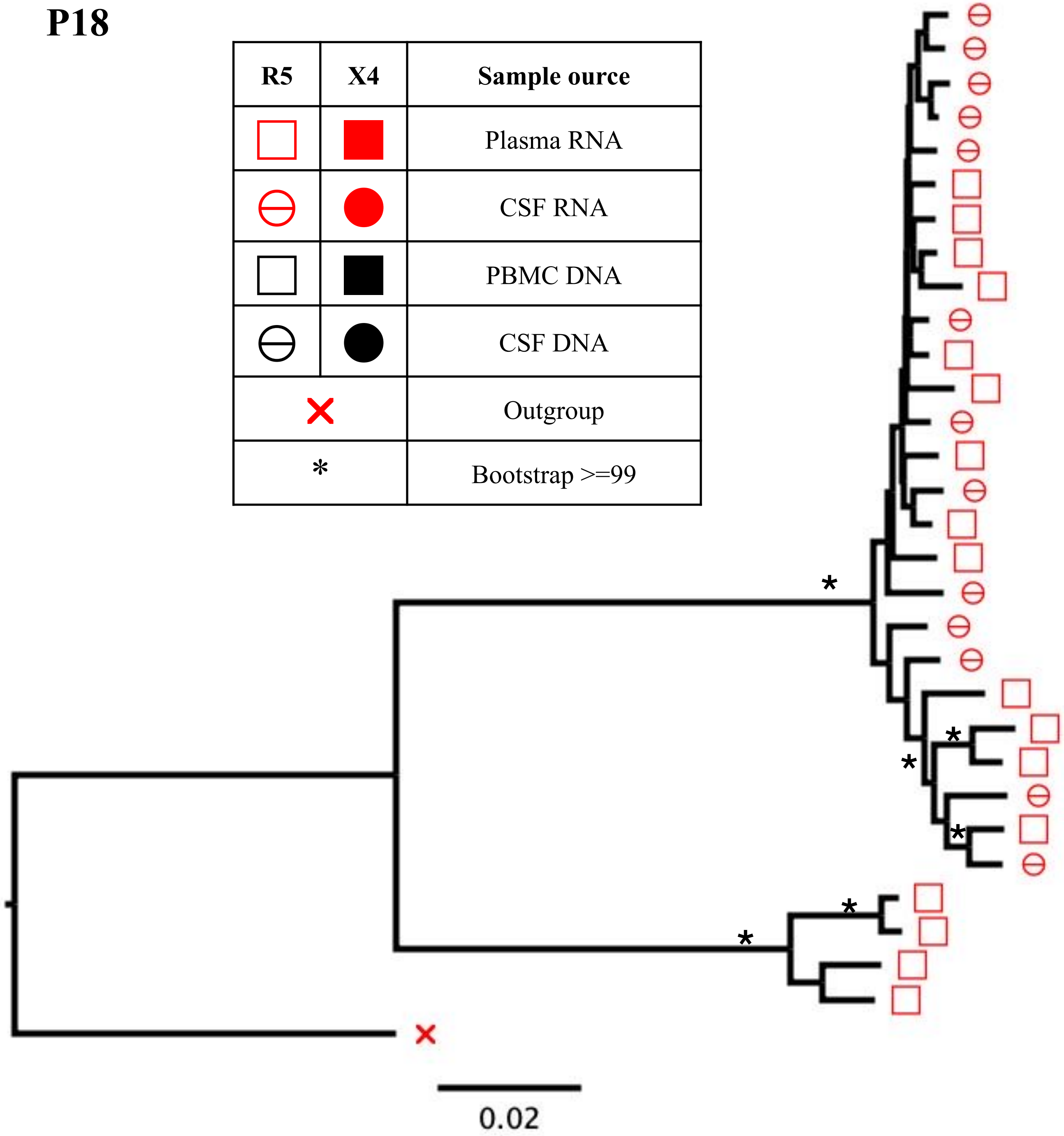
R5
X4
Sample ource
Plasma RNA
CSF RNA
PBMC DNA
CSF DNA
Outgroup
*
Bootstrap >=99
0.02

Tree of Trees (N=18)

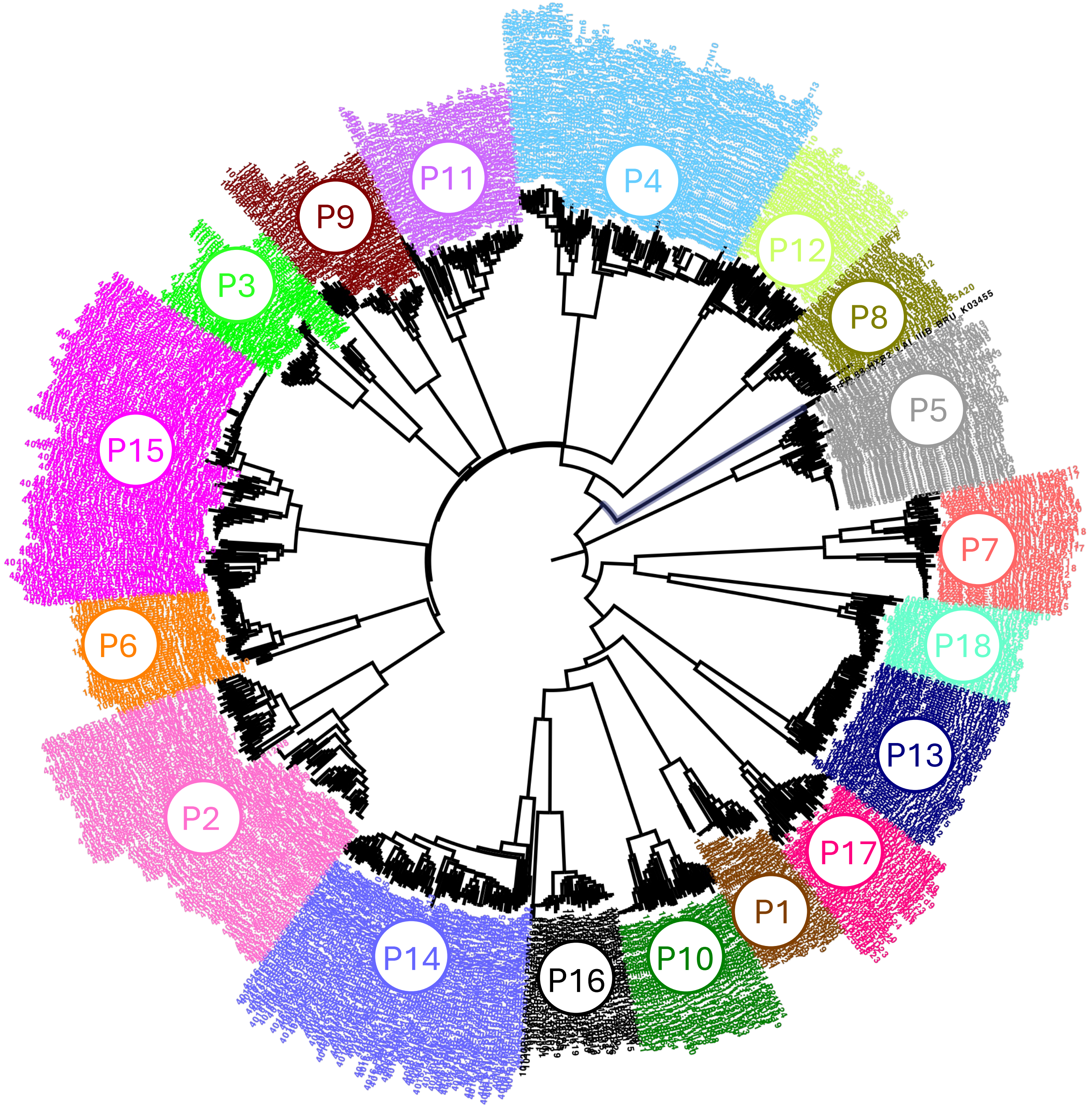

Supplement: Supplementary file 1 [file ppat.1014697.s013.pdf]
